# Chloroplast proteome response to drought stress and recovery in tomato (*Solanum lycopersicum* L.)

**DOI:** 10.1186/s12870-017-0971-0

**Published:** 2017-02-10

**Authors:** Rachele Tamburino, Monica Vitale, Alessandra Ruggiero, Mauro Sassi, Lorenza Sannino, Simona Arena, Antonello Costa, Giorgia Batelli, Nicola Zambrano, Andrea Scaloni, Stefania Grillo, Nunzia Scotti

**Affiliations:** 1 Institute of Biosciences and BioResources, National Research Council of Italy (CNR-IBBR), via Università 133, 80055 Portici, NA Italy; 2 Institute for the Animal Production System in the Mediterranean Environment, National Research Council of Italy (CNR-ISPAAM), via Argine 1085, 80147 Napoli, Italy; 3 Department of Molecular Medicine and Medical Biotechnology, University of Naples Federico II, via Pansini, 80100 Napoli, Italy; 4 Center of Genetics Engineering (CEINGE) Biotecnologie Avanzate S.c. a R.l, via Pansini, 80100 Napoli, Italy

**Keywords:** Water deficit, Proteomic analysis, Abscisic acid, Proline, Environmental sensor, Retrograde signaling

## Abstract

**Background:**

Drought is a major constraint for plant growth and crop productivity that is receiving an increased attention due to global climate changes. Chloroplasts act as environmental sensors, however, only partial information is available on stress-induced mechanisms within plastids. Here, we investigated the chloroplast response to a severe drought treatment and a subsequent recovery cycle in tomato through physiological, metabolite and proteomic analyses.

**Results:**

Under stress conditions, tomato plants showed stunted growth, and elevated levels of proline, abscisic acid (ABA) and late embryogenesis abundant gene transcript. Proteomics revealed that water deficit deeply affects chloroplast protein repertoire (31 differentially represented components), mainly involving energy-related functional species. Following the rewatering cycle, physiological parameters and metabolite levels indicated a recovery of tomato plant functions, while proteomics revealed a still ongoing adjustment of the chloroplast protein repertoire, which was even wider than during the drought phase (54 components differentially represented). Changes in gene expression of candidate genes and accumulation of ABA suggested the activation under stress of a specific chloroplast-to-nucleus (retrograde) signaling pathway and interconnection with the ABA-dependent network.

**Conclusions:**

Our results give an original overview on the role of chloroplast as enviromental sensor by both coordinating the expression of nuclear-encoded plastid-localised proteins and mediating plant stress response. Although our data suggest the activation of a specific retrograde signaling pathway and interconnection with ABA signaling network in tomato, the involvement and fine regulation of such pathway need to be further investigated through the development and characterization of ad hoc designed plant mutants.

**Electronic supplementary material:**

The online version of this article (doi:10.1186/s12870-017-0971-0) contains supplementary material, which is available to authorized users.

## Background

Drought stress represents a major constraint for agriculture worldwide causing significant yield losses and affecting crop quality [1]. Climate change phenomena are expected to increase frequency, intensity, and duration of drought episodes as well as to cause changes in the map of arid prone areas [2]. Therefore, stabilizing and/or improving plants performance under low water input represents a priority in plant science research with a wealth of information being lately accumulated on mechanisms of response at cellular, tissue and organ levels in model and crop species [3, 4].

Increasing evidence reveals a central role of chloroplast in plant stress response [5] and highlights the connection between different stress responses and organellar signaling pathways [6, 7]. Chloroplast is a semi-autonomous organelle since most plastid-localised proteins are nuclear-encoded. This implicates the existence of sophisticated communication mechanisms that allow adequate coordination of gene expression in both organelles, thus ensuring a correct functioning of overall cellular metabolism [3, 7–9]. Chloroplast harbours many cellular vital processes (i.e., aromatic amino acids, fatty acids and carotenoids biosynthesis and sulphate assimilation pathways) in addition to photosynthesis, and is considered a key element in plant stress response, because it acts as sensor of environmental changes optimizing different cell functions for triggering the adaptive response to stressful conditions. However, several aspects of plastid alterations following water deficit are still poorly characterized.

Under drought stress, reactive oxygen species (ROS) accumulation may be increased through multiple ways. For instance, the reduction of CO_2_ fixation, due to stomatal closure, leads to a reduced NADP^+^ regeneration through the Calvin cycle, which, in association with the changes in photosystem activities and photosynthetic transport capacity, results in a higher leakage of electrons to O_2_ with a subsequent increased production of ROS via the chloroplast Mehler reaction compared to unstressed plants [10]. Enhanced ROS levels result in peroxidation of lipids, oxidation of proteins and inhibition of enzymatic activities, oxidative damage to nucleic acids, and ultimately cell death.

Tomato (*Solanum lycopersicum* L.) is one of the most important crops worldwide. Over the last decade, its production increased continuously reaching almost 160 million tons fresh fruit in 2013 [11]. It is consumed as fresh or processed fruit due to its excellent nutritional properties, being a good source of vitamins, folate, and phytochemicals [12]. Tomato is also considered an ideal fleshy fruit model system, because it can be easily grown under different conditions, it has a short lifecycle, and simple genetics due to the relatively small genome and lack of gene duplication, etc. [13]. Furthermore, knowledge in tomato biology can be easily transferred to other economically important *Solanaceae* species [14]. Despite the economic relevance of this crop, the mechanisms underlying its response to abiotic stresses are not yet fully clarified and few information is currently available on key role of stress-responsive genes [15, 16].

Several genomic, proteomic or metabolomic studies have investigated the response to water deficit in crops, focusing on specific organs or the whole organism [4, 17–22]. Genomic studies clarified the role of specific genes in stress tolerance and identified promoters and *cis*-elements useful for crop engineering applications. In tomato, a recent study identified differentially expressed genes and corresponding enriched Gene Onthology (GO) categories after long-term drought stress and rehydration, showing that GOs enriched in down-regulated genes after drought stress included photosynthesis and cell proliferation. On the contrary, upregulated genes belonged to GO categories more directly connected to stress responses [23]. Proteomic studies highlighted specific changes in components involved in transcription/translation machineries and/or in structural elements regulating cytoplasm hydration [22, 24–26]. A proteomic study of drought stressed roots of *S. lycopersicum* and *S. chilense* identified several differentially accumulated proteins, with a majority in the down-regulated fraction in both genotypes. These belonged to categories related to cellular metabolic activities and protein translation [27]. Metabolomic analyses emphasized the accumulation of secondary metabolites involved in protection against water stress. For example, Rabara et al. [4] recently identified some of the metabolic changes of plants associated with water withholding, including the production of antioxidants (e.g., glutathione, tocopherol) and osmolytes as protective compounds against oxidative stress and for the regulation of the carbon/nitrogen balance, respectively. Further, accumulation, under prolonged water deficit, of phenolic derivatives in other species suggested the involvement of these compounds in the water stress adaptive response [28, 29].

Because the chloroplasts are central organelles where the photosynthetic reactions take place, modifications in their physiology and protein pools are expected in response to drought stress-induced variations in leaf gas exchanges and accumulation of ROS. The aim of the present study was to investigate the stress-induced mechanisms within plastids in response to a severe and prolonged water deficit and subsequent rewatering cycle in tomato combining comparative proteomic, molecular and physiological analyses. Our findings give an original overview on the significant role of chloroplast as environmental sensor by both coordinating the expression of nuclear-encoded plastid-localised proteins and mediating plant stress response.

## Methods

### Plant material and drought treatment

Three-weeks-old tomato (cv Crovarese) seedlings were obtained from seeds (kindly provided by La Semiorto Sementi, Italy) and transferred to flowerpots (48 L volume, Length × Width × Height = 55 × 30 × 25 cm) containing 16 L of a 1:3 mix of slightly/fully decomposed bog peat (pH 3.5-7): perlite, with a water holding capacity of 45% in volume. Plants were allowed to grow for 10 days in our research greenhouse (average day Temperature (T): 28 °C; average night T: 22 °C; Humidity 60%; Photoperiod: 15 h light/9 h darkness) in irrigated conditions. Subsequently, a subset of pots was subjected to drought stress by suspending irrigation for 19 days. A subset of drought stressed plants was allowed to recover by irrigating for 6 days. Plant status was monitored during the experiment by measuring stomatal conductance with an AP4 leaf porometer (Delta-T Devices, UK). After rewatering treatments, biometric parameters (e.g., number of nodes, height, total fresh and dry weight) were measured to assess the impact of water deficiency stress on tomato vegetative growth. In parallel, leaf samples from control (C), drought-stressed (D), control-recovered (CR) and drought-recovered (DR) plants were harvested for further biochemical and molecular analyses.

### Abscisic acid determination

ABA was quantified by competitive ELISA using the Phytodetek ABA test kit (Agdia, USA) following the manufacturer’s instructions and procedure described by Iovieno et al. [23]. Colour absorbance was measured at 405 nm using a plate auto reader (1420 Multilabel Counter Victor3TM, PerkinElmer, USA). Three biological and three technical replicates were used to evaluate each experimental condition.

### Proline determination

Proline content was determined according to the method of Claussen [30] as previously described [23]. Three biological and three technical replicates were used to evaluate each experimental condition.

### Pigments determination

Chlorophylls (a and b) and carotenoids were extracted from leaves of tomato plants (0.1 g) with 96% *v/v* ethanol containing 0.3% *w/v* NaHCO_3_, as described by Lichtenthaler [31]. Pigments content was spectrophotometrically determined at 665, 649 and 470 nm using a Perkin Elmer Lambda25 UV/VIS spectrometer. Calculations were based on formulas described elsewhere [32]. Three biological and three technical replicates were used to evaluate each experimental condition.

### Chloroplast isolation

Pure and intact chloroplasts were isolated from about 30 g of tomato leaves, as previously described [33] with minor modifications. All steps were carried out at 4 °C. Fresh tomato leaves were homogenized in 20 mM Tricine-KOH (pH 8.4), 10 mM EDTA, 10 mM NaHCO_3_, 0.45 M sorbitol and 0.1% *w/v* BSA, twice, for 5 s, with a Waring Blendor operating at high speed. Homogenates were filtered through 4 layers of muslin and one layer of Miracloth (GE Healthcare, UK), and centrifuged at 2100 × *g*, for 5 min. Pellets were resuspended in washing buffer (18 ml) and then loaded onto a continuous percoll gradient (Sigma-Aldrich, USA), which was centrifuged at 13,000 × *g*, for 10 min. Intact chloroplasts were carefully recovered and resuspended in 5 vol of 20 mM Tricine-KOH (pH 7.6), 5 mM MgCl_2_, 2.5 mM EDTA and 0.3 M sorbitol. After centrifugation at 2100 × *g* for 5 min, pellets were recovered and freezed in liquid N_2_. Chloroplasts were stored at – 80 °C. Three biological replicates were used for each experimental conditions either for challenged and control plants.

### Plastid protein extraction and 2D-DIGE analysis

Chloroplast proteins from three biological replicates were extracted according to a modified borax/PVPP/phenol (BPP) protocol [34]. Briefly, isolated chloroplast pellets were resuspended in a buffer containing 100 mM Tris, 100 mM EDTA, 50 mM borax, 50 mM vitamin C, 1% *w/v* Triton X-100, 2% *w/v* β-mercaptoethanol, 30% *w/v* sucrose and protease inhibitors and vortexed for 5 min, at room temperature. An equal volume of Tris-saturated phenol (pH 8.0) was added to samples, which were then vortexed for 10 min and centrifuged at 15,000 × *g*, for 15 min, at 4 °C; corresponding upper phenol phases were transferred to novel centrifuge tubes. Proteins were precipitated by adding 5 vol of ammonium sulphate-saturated methanol and incubated at −20 °C, for 16 h. After centrifugation at 15,000 × *g*, for 15 min, at 4 °C, protein pellets were washed with ice-cold methanol once and with ice-cold acetone three times. After each wash, protein pellets were centrifuged at 15,000 × *g*, for 15 min, at 4 °C, and the supernatant was decanted. Finally, the pellets were dissolved in 30 mM Tris–HCl, 7 M urea, 2 M thiourea and 4% CHAPS. Protein concentration was determined using the Bradford method (Bio-Rad, USA); the pH value of each sample was adjusted to pH 8.5 with HCl. Fifty microgram of proteins belonging to each experimental condition were labelled with 400 pmol of Cy2-, Cy3- or Cy5-dyes (GE Healthcare, UK), using a dye-swapping strategy. Six mixtures of the 12 samples were labelled with Cy2 dye, as the internal standard required by the 2D-DIGE protocol. Each labelling reaction was performed in the dark for 30 min at 0 °C, and quenced with 1 mM lysine. Appropriate Cy3- and Cy5-labeled pairs and a Cy2-labeled control were used to generate mixtures. Each mixture was supplemented with 1% *v/v* IPG buffer, pH 3-10 NL (GE Healthcare, UK), 1.4% *v/v* DeStreak reagent (GE Healthcare, UK) and 0.2% *w/v* DTT to reach a final volume of 450 μl in 7 M urea, 2 M thiourea, and 4% *w/v* CHAPS. The mixtures (150 μg of total protein content) were used for passive hydration of immobilized pH gradient IPG gel strips (24 cm, pH 3-10 NL) in the dark, for 16 h, at 20 °C. Isoelectric focusing (IEF) was carried out with an IPGphor II apparatus (GE Healthcare) up to 80,000 V/h at 20 °C. After IEF, each strip was equilibrated with an equilibration solution composed of 6 M urea, 2% *w/v* SDS, 20% *w/v* glycerol, and 0.375 M Tris–HCl (pH 8.8), in the presence of 0.5% *w/v* DTT, for 15 min, in the dark; then, it was equilibrated in the same buffer containing 4.5% *w/v* iodacetamide, for another 15 min. Equilibrated IPG strips were transferred onto 12% polyacrilamide gels to perform the second-dimension SDS-PAGE, using an ETTAN DALT six electrophoresis system (GE Healthcare, UK). Gels were scanned with a Typhoon 9400 variable mode imager (GE Healthcare, UK) using proper excitation/emission wavelengths for Cy2 (488/520 nm), Cy3 (532/580 nm), and Cy5 (633/670 nm). Gel images were visualized with the Image-Quant software (GE Healthcare, UK) and analyzed using the DeCyder 6.0 software (GE Healthcare, UK). A DeCyder differential In-gel-Analysis (DIA) module was used for spot detection and pairwise comparison of each sample (Cy3 and Cy5) to the Cy2 mixed standard present in each gel. Then, the DeCyder Biological Variation Analysis (BVA) module was used to simultaneously match all of the protein-spot maps from the gels, and to calculate average abundance ratios and statistical parameters (Student’s *T*-test). Differentially represented spots were identified as those having a relative expression ratio >1.50 or <1.50, with a *P* value ≤ 0.05.

Preparative two-dimensional gel electrophoresis was performed using 500 μg of unlabeled protein samples. The resulting 2D-PAGE gels were stained with Sypro Ruby protein gel stain, according to the manufacturer’s instructions (Thermo Fisher Scientific, USA). After spot matching with the master gel from the analytical assay in the BVA module of DeCyder software, a pick list was generated for spot picking by a robotic picker (Ettan spot picker, GE Healthcare, UK).

### Protein identification by mass spectrometry

Spots from 2D-DIGE were triturated, washed with water, *in-gel* reduced with DTT, S-alkylated with iodoacetamide, and then digested with trypsin. Resulting peptide mixtures were desalted by μZip-TipC_18_ using 50% *v/v* acetonitrile, 5% *v/v* formic acid as eluent. Recovered peptides were then analyzed for protein identification by nLC-ESI-LIT-MS/MS, using an LTQ XL mass spectrometer (Thermo Fisher Scientific, USA) equipped with a Proxeon nanospray source connected to an Easy-nanoLC (Proxeon, Denmark) [35]. Peptides were resolved on an Easy C18 column (100 mm × 0.075 mm, 3 μm) (Proxeon). Mobile phases were 0.1% *v/v* formic acid (solvent A) and 0.1% *v/v* formic acid in acetonitrile (solvent B), running at a total flow rate of 300 nL/min. Linear gradient was initiated 20 min after sample loading; solvent B ramped from 5 to 35% over 45 min, from 35 to 60% over 10 min, and from 60 to 95% over 20 min. Spectra were acquired in the range *m/z* 400–2000. Each peptide mixture was analyzed under collision-induced dissociation (CID)-MS/MS data-dependent product ion scanning procedure, enabling dynamic exclusion (repeat count 1 and exclusion duration 60 s), over the three most abundant ions. Mass isolation window and collision energy were set to *m/z* 3 and 35%, respectively.

Raw data from nLC-ESI-LIT-MS/MS analysis were searched by MASCOT search engine (version 2.2.06, Matrix Science, UK) against an updated (2014/05/06) NCBI non-redundant database (taxonomy *Solanum lycopersicum*) in order to identify proteins from gel spots. Database searching was performed by using Cys carbamidomethylation and Met oxidation as fixed and variable protein modifications, respectively, a mass tolerance value of 1.8 Da for precursor ion and 0.8 Da for MS/MS fragments, trypsin as proteolytic enzyme, and a missed cleavage maximum value of 2. Other MASCOT parameters were kept as default. Protein candidates assigned on the basis of at least two sequenced peptides with an individual peptide expectation value <0.05 (corresponding to a confidence level for peptide identification >95%) were considered confidently identified. Definitive peptide assignment was always associated with manual spectra visualization and verification.

### SDS-PAGE and western blot analysis

Total soluble leaf proteins were extracted from C, D, CR and DR plants according to Petersen and Bock [36], and resolved by electrophoresis on either 10 or 15% SDS-PAGE. Gels were blotted onto nitrocellulose membranes (Hybond^TM^-ECL^TM^, Amersham Biosciences, UK) at constant voltage (100 V), for 90 min. Membranes were incubated in blocking buffer (TBS containing 0.1% *v/v* Tween and 5% *w/v* defatted milk), for 1 h at room temperature. Membranes were challenged with primary antibodies (PsbP6 AS06 167; PsbQ AS06 142-16; GS2 AS08 296; FNR AS10 1625; RA AS10 700; RbcL AS03 037; ATP synthase β AS05 085; CSP41b AS08 298; light harvesting complex Lhcb1 AS09 522; elongation factor EF1α AS10 934 - Agrisera, Sweden), which were diluted in blocking buffer according to manufacturer’s instructions, and used at 4 °C overnight. A HRP-conjugated anti-rabbit antibody (1:60,000 dilution, GE Healthcare, UK) was applied for 1 h, at room temperature. Detection was carried out with ECL (GE Healthcare) following manufacturer’s instructions. Chemiluminescent signals were measured using a ChemiDoc^TM^ XRS+ (Bio-Rad) and images were analysed by using the Image Lab^TM^ Software (Bio-Rad). The protein accumulation levels are the mean of three biological samples presented with SD values. Significant differences were analysed by Student’s *t* test (^*^ *P* ≤ 0.05, ^**^ *P* ≤ 0.01).

### qRT-PCR

Total RNA from leaf samples was extracted with the RNeasy® Plant Mini kit (Qiagen, Germany). cDNA was synthetized using the QuantiTect® Reverse Transcription kit (Qiagen) following the manufacturer’s instructions. Tomato orthologous genes were identified through Sol Genomics Network website [37] by using *A. thaliana* sequences obtained through TAIR website [38]. Gene-specific qRT-PCR primers (Additional file 1: Table S1) were designed using the online software Integrated DNA Technologies (IDT). The mixed solution of qRT-PCR reaction contained Platinum® SYBR® Green qPCR SuperMix-UDG with ROX (2×, Invitrogen, USA), reverse and forward primers mix (4.28 μM) and 20-fold-diluted cDNA template. All reactions were performed on a 7900HT Fast Real-Time PCR system (Applied Biosystems, USA). Reaction conditions were 10 min at 95 °C, followed by 40 cycles of heating at 95 °C and annealing at 60 °C for 15 and 60 s, respectively. To verify single-product amplification, melting curves were carried out in each PCR. The relative level of gene expression was calculated with 2^- (ΔΔCt)^ algorithm; EF-1α was used as internal control [39]. Three biological samples and two technical replicates for each experimental condition were analysed. The significant differences of expression level between D and DR and their corresponding control samples were evaluated using Student’s *t* test (^*^ *P* ≤ 0.05, ^**^ *P* ≤ 0.01).

### Statistical analysis

Statistical analysis was performed on three biological replicates for biochemical (i.e., ABA, proline, chlorophyll and carotenoid contents) and molecular (i.e., protein and transcript levels determination) analyses. Stomatal conductance measurements were carried out using six biological replicates for C, CR and DR and nine for D, respectively; biometric measurements were performed with 12 biological samples for CR and 30 for DR, respectively. Data were given as means ± standard deviation (SD). Significant differences between drought-stressed and drought-recovered samples and their corresponding controls for all measurements described were analysed by an unpaired two-tailed Student’s *t* test (^*^ *P* ≤ 0.05, ^**^ *P* ≤ 0.01). Statistical analysis of gel spot quantitative differences was carried out as reported in the dedicated section, using the Student’s *t* test.

## Results

### Morphological, physiological and biochemical responses to drought stress

**Fig. 1:**
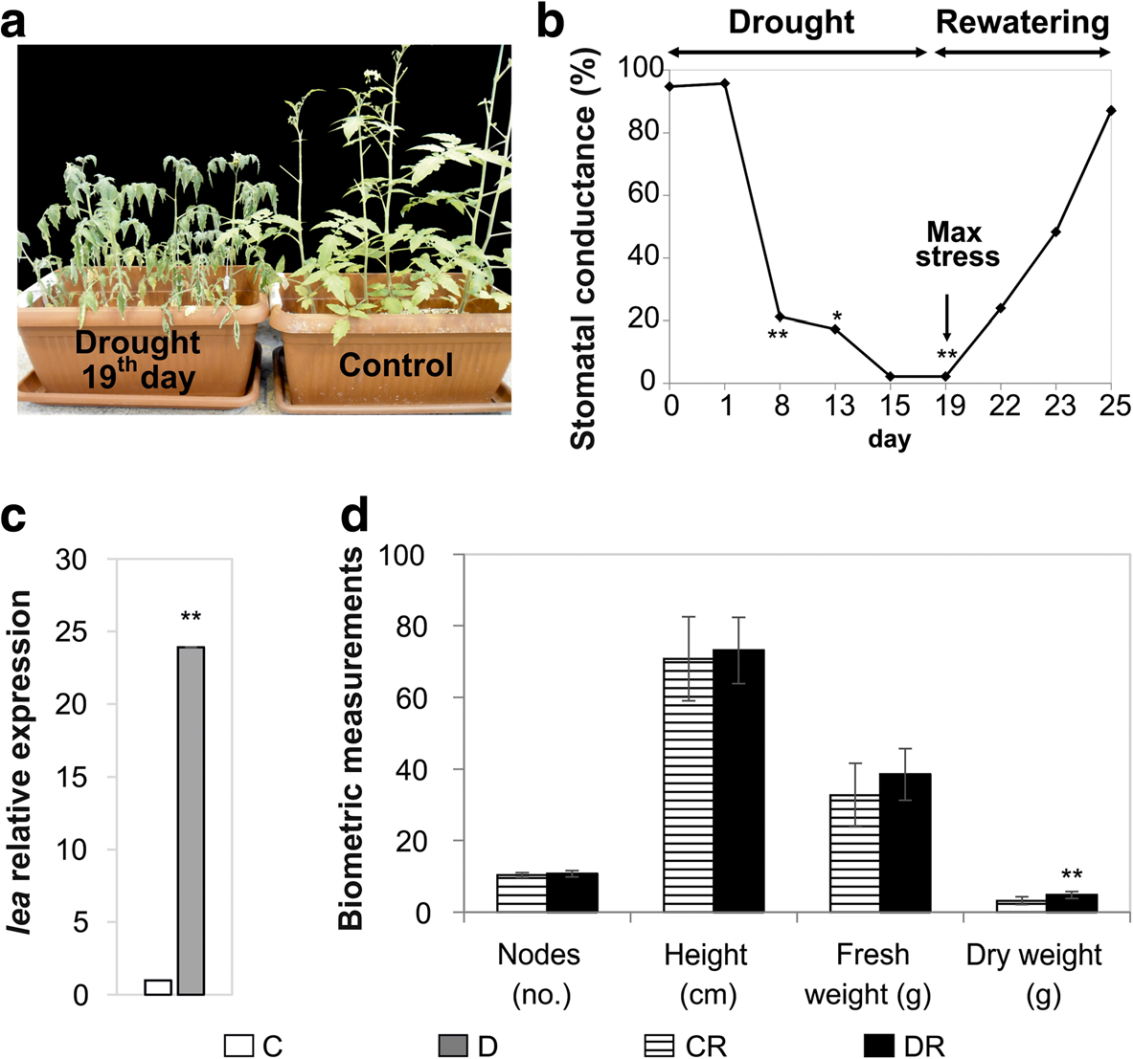
Morphological and physiological responses to drought-stress. **a** Tomato plants at the 19th day of water withholding (*Left*) compared to control plants of the same age (*Right*). **b** Stomatal conductance of drought-stressed plants expressed as percentage of corresponding values of control plants of the same age. The data represent the mean of six biological replicates for C and DR and nine for D. **c** qRT-PCR analysis of late embryogenesis abundant gene (*lea*), which was used as stress-marker gene. The housekeeping *elongation factor* (EF-1α) gene was used for normalization; *error bars* indicate SD, *n* = 3. **d** Biometric measurements of control-recovered and drought-recovered plants. Measurements were conducted at the end of the rewatering treatment. Reported values are the mean ± SD of 12 biological samples for CR and 30 for DR (^*^ *P* ≤ 0.05, ^**^ *P* ≤ 0.01). *C* control, *D* drought-stressed, *CR* control-recovered, *DR* drought-recovered plants

A long-term drought treatment was applied to tomato plants by water withholding for 19 days followed by a rewatering phase. Plants grown under drought conditions showed severe wilting symptoms and a stunted growth when compared to control plants, thus indicating a water stress status (Fig. 1a). During the experiment, several parameters were measured to monitor plant stress. As shown in Fig. 1b, stomatal conductance decreased continuously reaching a value corresponding to 2% of that of control plants after 19 days of water withholding. Furthermore, the prolonged drought conditions activated the expression of a known stress-responsive gene Solyc03g116390.2.1 [15], encoding a late embryogenesis abundant (Lea) protein, (Fig. 1c). After a cycle of rewatering (6 days), stressed plants showed a nearly complete recovery, as revealed by stomatal conductance measurements (Fig. 1b), and other biometric parameters (i.e., number of nodes, height, total fresh and dry weight), which were comparable to those of control plants (Fig. 1d).

**Fig. 2:**
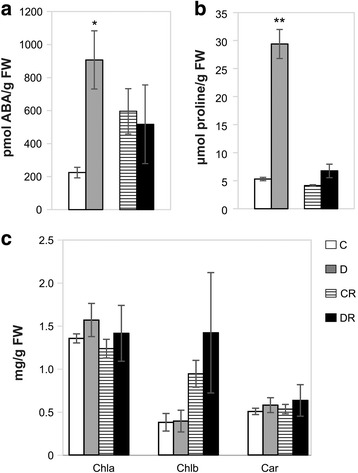
Metabolite content in the leaves of the drought-stressed and drought-recovered plants, compared to corresponding controls. (**a**) Abscisic acid (ABA); (**b**) Proline; (**c**) Chlorophyll a (Chla), chlorophyll b (Chlb) and carotenoids (Car). Values are the mean ± SD of three biological replicates (^*^ *P* ≤ 0.05, ^**^ *P* ≤ 0.01). *FW* Fresh Weight, *C* control, *D* drought-stressed, *CR* control-recovered, *DR* drought-recovered plants

To evaluate metabolic changes in response to stress, levels of the phytohormone abscisic acid (ABA) and the compatible osmolyte proline (Pro), were quantified. In drought stressed plants, ABA and Pro levels increased up to 5- and 6-fold, respectively (Fig. 2a and b). As expected, at the end of rewatering phase the content of such metabolites was similar to that of control plants (Fig. 2a and b). No significant variations in chlorophylls and carotenoids content were observed in response to water deficit conditions and after rewatering (Fig. 2c).

### Chloroplast proteome

In order to investigate the response of chloroplasts to water deficit and the following rewatering cycle in tomato, a comparative proteomic analysis based on combined two-dimensional difference *in gel* electrophoresis (2D-DIGE) and nano-liquid chromatography coupled with electrospray ionization-linear ion trap tandem mass spectrometry (nLC-ESI-LIT-MS/MS) experiments was performed on plastid extracts from C, D, CR and DR plants. The corresponding 2D-maps were characterized by the occurrence of about 2600 protein spots in the pH 3–10 range, with molecular mass values of 10–120 kDa (Additional file 1: Figure S1). Image analysis revealed those spots having at least a 1.5-fold change in relative abundance with respect to corresponding control samples (*P* ≤ 0.05). In particular, it showed that drought treatment determined a differential representation of 57 protein spots, among which 28 were over-represented and 29 down-represented (Additional file 1: Table S2). Similarly, plastids from plants recovered after drought showed 148 differentially represented spots when compared to well-watered control plants of the same age; among that, 19 were over-represented and 129 down-represented, respectively (Additional file 1: Table S2). Differentially represented spots (201 in number) plus selected constant spots (22 in number) were digested with trypsin, and then analysed by nLC-ESI-LIT-MS/MS for protein identification. Identification details and statistics are reported in Additional file 2: Table S3.

Differentially represented spots in D and DR plants were associated with 31 and 54 sequence entries, respectively (Additional file 1: Table S2). In most cases (89%), a unique protein species occurred within the variably represented spot, probably as a result of the limited number of components present in the chloroplast sample after organelle subfractionation. When multiple components comigrated within the same spot, they were often associated with protein species differing for few amino acid substitutions. When this was not the case, protein quantitative information was easily inferred by comparison with data from other vicinal spots. In fact, all the identified proteins showed a coherent quantitative trend among the experimental conditions; exceptions were transketolase (TK), ribulose-1,5-bisphosphate carboxylase/oxygenase large subunit (RbcL), phosphoribulokinase (PRK), chloroplast sedoheptulose-1,7-bisphosphatase (SBPase), ascorbate peroxidase (Apx-TL29), oxygen-evolving enhancer protein 1 (OEE1) and 33 kDa precursor protein of oxygen-evolving complex (OEC1). These proteins presented an either constant, over- and down-representation under the same experimental condition, probably as result of post-translational modification events. Finally, detection of the auxin binding protein (ABP19α-like), which occurs in plant membranes and ER [40], and of the mitochondrial Clp protease 2 [41] demonstrated a minimal contamination of the chloroplast preparations with extraplastidic subfractions (Additional file 1: Table S2).

**Fig. 3:**
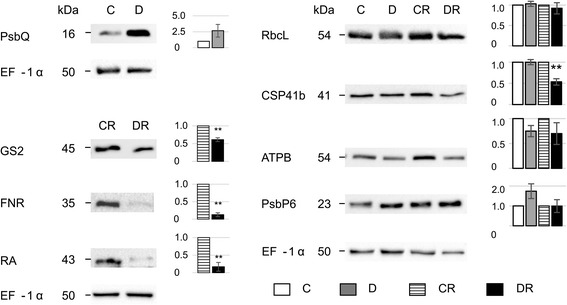
Representative western blot validation of selected differentially regulated proteins as detected by proteomic experiments. Equal protein loading was confirmed with the anti-elongation factor (EF-1α) antibody. Histograms (on the *right*) represent relative protein abundance in C, D, CR and DR plants. *C* control, *D* drought-stressed, *CR* control-recovered, *DR* drought-recovered plants. Protein levels are mean ± SD, *n* = 3 (^**^ *P* ≤ 0.01)

Proteomic data were validated by western blot analysis on selected components, based on limited commercial availability of antibodies for chloroplast proteins (Fig. 3). Overall, western blotting experiments confirmed 2D-DIGE results. In particular, photosystem II oxygen-evolving complex protein 3 (PsbQ) resulted over-represented up to 2.5-fold in D compared to C plants. Analogously, glutamine synthase (GS2), ferredoxin-NADP reductase (FNR) and ribulose-1,5-bisphosphate carboxylase/oxygenase activase 1 (RA) were down-represented in DR plants, resulting 0.6, 0.1 and 0.17-fold compared to controls, respectively. Chloroplast stem-loop binding protein 41 kDa (CSP41b) and ATP synthase β (ATPB) were slightly down-represented in D and DR plants, although their levels dropped down after the rewatering cycle, being about 0.5 and 0.7-fold of controls, respectively. Further, PsbP domain-containing protein 6 (PsbP6) was slightly over-represented of 1.7 and 1.2 fold in D and DR plants compared to corresponding controls, respectively (Fig. 3). Finally, RbcL levels were unaltered in D plants and slightly down-represented after rewatering. Quantitative differences observed in the latter case with respect to 2D-DIGE results were ascribed to the variable representation of the RbcL spots within the gels (see above).

**Fig. 4:**
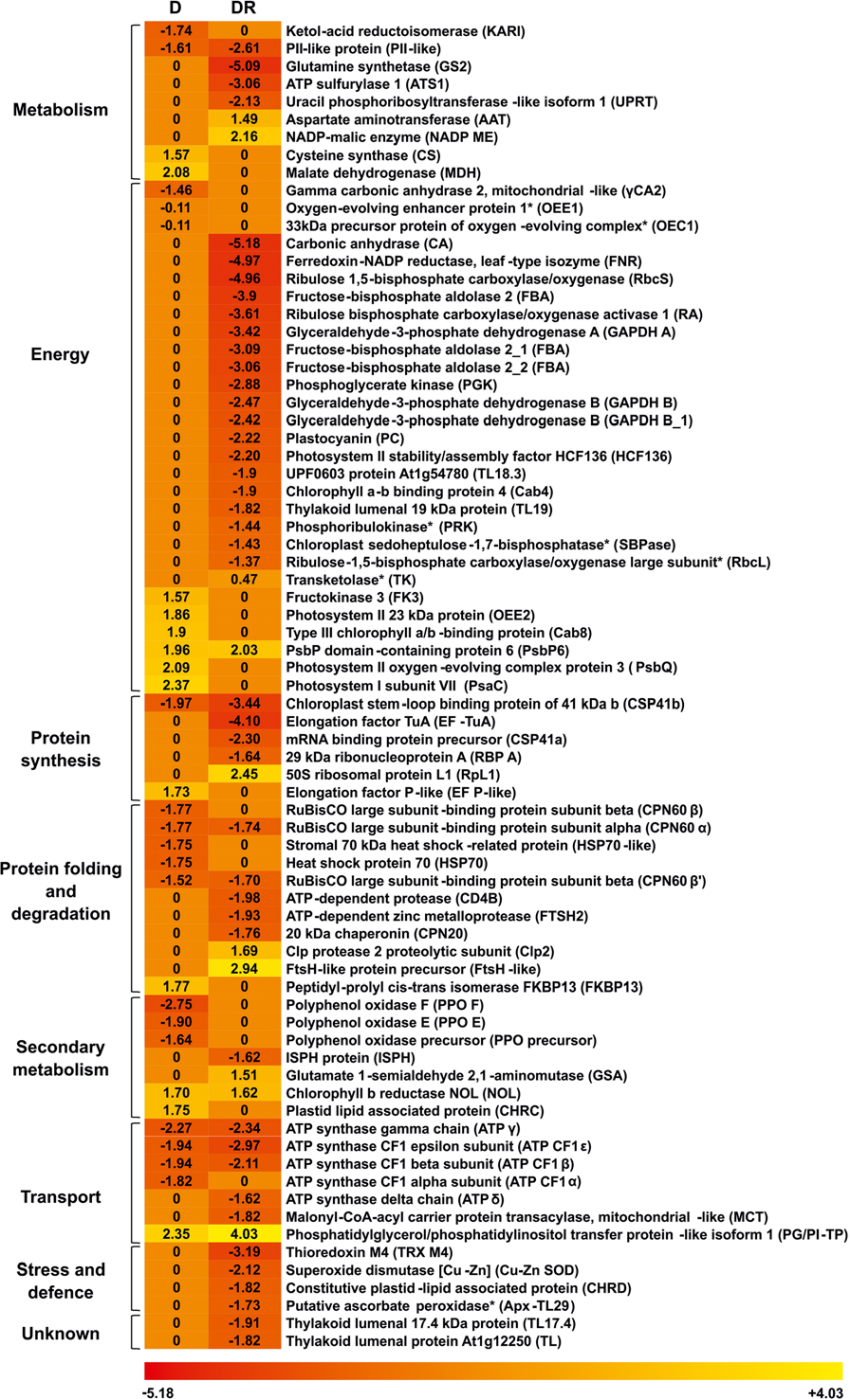
Differentially represented plastid proteins in tomato chloroplasts after drought stress and following a subsequent rewatering cycle. The *color scale bar* indicates increased levels (*yellow*), decreased levels (*red*), or no significant changes (*orange*). *Asterisks* indicate proteins showing an incoherent quantitative trend among the experimental conditions. Reported values refer to the mean fold change of the various spots corresponding to each protein, as compared to corresponding controls (see Additional file 1: Table S2 for individual spot changes). Putative functional categories are indicated on the *left. D* drought-stressed, *DR* drought-recovered plants

In order to rationalize protein families most affected by water deficit, differentially represented plastid proteins were grouped into categories, based on their known or predicted functions [42] (Table 1 and Fig. 4). As expected, photosynthesis was the process most affected at the peak of drought stress (19th day), accounting for 29% of the regulated components identified in this study. It was included in the *energy* category and it was mainly represented by components of both photosystems (e.g., PsaC, PsbP6, PsbQ, etc.). The second most abundant (19.3%) category of regulated proteins was that of species involved in *protein folding and degradation*, followed by *transport* category (16.1%), which mainly included ATP synthase constituents and other transporters.

**Table 1:** Functional classification of the proteins differentially represented in drought-stressed and drought-recovered plants, compared to their respective controls. Number of proteins belonging to indicated functional categories are reported together with the percentage over the total number of differentially represented proteins

Functional category	Drought-stressed	Drought-recovered
Metabolism	4 (12.9%)	6 (11.1%)
Energy^a^	9 (29.0%)	21 (38.9%)
Protein synthesis	2 (6.4%)	5 (9.3%)
Protein folding and degradation	6 (19.3%)	7 (13.0%)
Secondary metabolism	5 (16.1%)	3 (5.6%)
Transport	5 (16.1%)	6 (11.1%)
Stress and defence	-	4 (7.4%)
Unknown	-	2 (3.7%)
Total	31	54

^a^The ‘energy’ category includes photosynthesis

After plant rehydration, a larger number of proteins was altered in their relative quantitative representation, thus indicating an adjustment of the corresponding plastid proteome (Fig. 4). Similarly to D plants, photosynthesis was again observed as the protein family most impaired after rehydration (39% of the regulated proteins). In this case, it was mainly represented by proteins involved in Calvin-Benson cycle (e.g., ribulose 1,5-bisphosphate carboxylase/oxygenase small and large subunits, sedoheptulose 1,7-bisphosphatase, etc.). Further, *protein folding and degradation* and *transport* categories were highly altered following the rewatering cycle, accounting for 13 and 11% of the regulated proteins, respectively.

### Correlation of gene expression with protein abundance

**Fig. 5:**
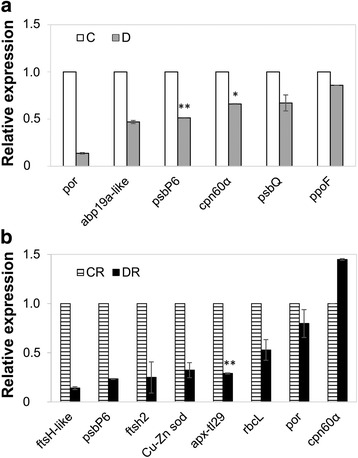
Expression profile of genes encoding differentially represented plastid proteins in drought stressed (**a**) and drought-recovered (**b**) plants, as deduced from proteomic analysis. Transcript level was determined by qRT-PCR after normalization to the elongation factor1-α (EF-1-α) gene as 2^−ΔΔCt^. *Error bars* indicate SD, *n* = 3. Significant differences were analysed by Student’s *t* test (^*^ *P* ≤ 0.05, ^**^ *P* ≤ 0.01). *C* control, *D* drought-stressed, *CR* control-recovered, *DR* drought-recovered plants

To verify whether the changes in protein abundance detected by 2D-DIGE were due to a regulation at translational or transcriptional level, a qRT-PCR analysis was carried out on RNA extracted from C, D, CR and DR samples (Fig. 5). Proteins, whose corresponding transcripts were evaluated, were selected among those encoded by genes (Additional file 1: Table S2) present in single copy in the tomato genome, which were also identified as differentially expressed in another tomato cultivar subjected to drought or rewatering conditions [23]. Among the analysed genes, those corresponding to Rubisco large subunit-binding protein subunit alpha (*cpn60α*) in D plants, and ascorbate peroxidase (TL29) (*apx-tl29*), superoxide dismutase (*Cu-Zn sod*), ATP-dependent zinc metalloprotease FTSH2 (*ftsH2*), protochlorophyllide reductase (*por*), and ribulose-1,5-bisphosphate carboxylase/oxygenase large subunit (*rbcL*) in DR plants showed a conserved trend compared to protein abundance (Fig. 5). By contrast, gene expression of auxin binding protein (*abp19a-like*), *por*, *psbP6* and *psbQ* in D plants, and of *ftsH-like*, *psbP6* and *cpn60α* in DR plants showed a different profile compared to corresponding proteins. Finally, polyphenol oxidase F (*ppoF*) transcript abundance was unaltered compared to controls in D plants (Fig. 5).

### Chloroplast-to-nucleus communication in tomato plants

**Fig. 6:**
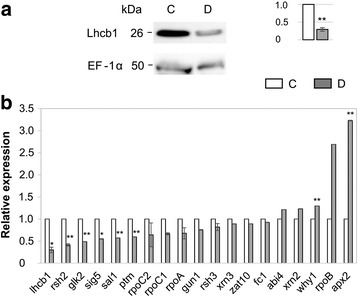
Chloroplast-to-nucleus communication analysis. **a** Western blot analysis of Lhcb1 protein; equal protein loading was verified by elongation factor (EF-1α) measurements. Histogram (on the *right*) reports the expression level of Lhcb1 in drought-stressed plants normalized to their control. **b** Expression of representative nuclear-encoded genes already known as being directly or indirectly involved in retrograde signaling pathways. Transcript level was determined by qRT-PCR after normalization to the elongation factor1-α (EF-1-α) gene as 2^−ΔΔCt^. *Error bars* indicate SD, *n* = 3. Significant differences were analysed by Student’s *t* test (^*^ *P* ≤ 0.05, ^**^ *P* ≤ 0.01). *C* control, *D* drought-stressed, *CR* control-recovered, *DR* drought-recovered plants

Chloroplasts are semi-autonomous organelles that communicate with nucleus to adjust gene expression in response to their requirements. In order to identify potential retrograde signaling pathways (plastid-to-nucleus communication) in tomato that are regulated by nuclear gene expression in response to stress, we investigated by qRT-PCR the expression of selected nuclear-encoded orthologous genes (Additional file 1: Table S1) already identified as associated with these biogenic and operational events in *A. thaliana* [43, 44]. As a first indication of the activation of retrograde signaling by our stress conditions, we monitored by western blotting and qRT-PCR the light harvesting complex protein (Lhcb1) and the corresponding gene (*lhcb1*) transcript, which is a favoured marker of retrograde signaling and is generally repressed under stress conditions [45, 46]. Analyses revealed a statistically significant down-representation of both protein and transcript abundance (about 70%) in D compared to C plants (Fig. 6a and b). In contrast, most of the genes (e.g., *fc1*, *gun1*, *xrn2*, *xrn3*, etc.) involved in chloroplast-to-nucleus communication in *A. thaliana* mutants did not show a marked variation in tomato D plants. However, noteworthy is the down-regulation of 3′(2′),5′-bisphosphate nucleotidase (*sal1*) gene, whose protein product degrades 3′-phosphoadenosine 5′-phosphate (PAP), a drought-induced metabolite involved in the expression of stress-responsive genes [47]. On the other hand, a statistically significant increase (about 3-fold) was observed for the cytosolic ascorbate peroxidase-coding gene (*apx2)*, which is involved in ROS scavenging (Fig. 6b).

## Discussion

Plant response to drought is a very complex process involving changes at physiological and biochemical levels, which can vary depending on the crop type and the age of plants as well as the duration and the severity of stress. Since plastids are essential for metabolism and environmental sensing, a detailed knowledge of the changes induced in this organelle in response to drought is essential to understand the mechanisms underlying plant adaptation to stress [5]. In this study, we have focused on the chloroplast response to a long-term drought stress and following a recovery cycle, and monitored drought stress and recovery progression measuring physiological and biochemical parameters.

In response to water deficit, we observed a slower plant development and a rapid decrease of the leaf stomatal conductance up to an almost complete stomata closure at the maximum point of stress (Fig. 1a and b), which corresponded to the accumulation of the plant stress metabolic markers ABA and Pro (Fig. 2a and b). Similar results were described for several species subjected to dehydration [4, 23, 48]. The enhanced accumulation of the *lea* gene transcript we observed was in accordance with a cell dehydration status of tomato (Fig. 1c) and with previous observations on other plants subjected to water deficit conditions [49, 50].

It is well known that water deprivation accelerates the degradation of photosynthetic pigments due to the deterioration of thylakoid membranes [51, 52]. Despite the stress condition, tomato plants did not significantly change their chlorophylls content (Fig. 2c), as observed in other drought-treated species [51]. An analogous trend was detected for carotenoids, photoprotective molecules involved in excess energy dissipation [53], as demonstrated in drought-stressed maize seedling [54]. It was suggested that plants maintaining high levels of photosynthetic pigments under water deficit are able to use light energy more efficiently than others and show an increased drought resistance [52]. Thus, the stable quantity of pigments detected in this study suggests that the tomato cultivar investigated has a robust tolerance to drought. Chloroplast proteome was highly affected by water deficit and proteins involved in photosynthetic process were mostly altered suggesting that photosynthesis was the main cellular process influenced (Fig. 4 and Table 1). Photosynthesis is a tightly regulated process that needs the coordinated coupling of light/dark reactions [3] and plays a central role in modulating energy signaling and balance [55]. The increase in relative abundance of photosystem components (PsaC, PsbP6 and PsbQ) suggests the attempt of tomato plants to maintain energy homeostasis. Particularly, over-representation of PsaC was indicative of the stimulation of the PSI-driven cyclic electron flow, process demonstrated to be induced by drought that allows the thermal dissipation of the energy excess [56–59].

ATP synthase activity is strictly related to photosynthesis because it transfers protons through the thylakoid membrane. About a 2-fold reduction was detected for several subunits of the ATP synthase complex (e.g., ATP α, β, γ and ε) after drought treatment, indicating a regulation of the whole machinery. A decrease of the ATP synthase activity was demonstrated to mediate non-photochemical quenching that protects photosynthetic apparatus from photo-damage as already observed in several species under stressful conditions [60–63]. According to this interpretation was the over-representation of peptidyl-prolyl *cis-trans* isomerase FKBP13, which has been reported to assist the assembling of photosystem subunits for energy balance [64]. FKBP13 interacts with the Rieske protein, an essential component of the cytochrome b_6_f complex in the photosynthetic electron-transfer chain, and acts as an anchor chaperone avoiding the excessive accumulation of this protein into the thylakoid [65]. An opposite quantitative trend was observed for others proteins belonging to the *folding and protein degradation* category (Cpn60α, Cpn60β, HSP70, etc.). As recently summarized by Kosova et al. [66], contrasting results about the accumulation of protein chaperones in response to drought have been reported in different plant species.

Upon drought stress, an alteration of the carbon/nitrogen ratio is expected as result of CO_2_ limitation, with consequent nutrient mobilization. Indeed, a 2-fold increase was observed for malate dehydrogenase (MDH), which participates in the Krebs cycle and has a key role in the regulation of carbon/nitrogen ratio. Similar results were reported in drought-treated barley, wild watermelon, rapeseed and wheat [20, 67]. MDH over-representation can also be associated with the regeneration of the electron acceptor NADP^+^ in chloroplasts, when CO_2_ assimilation is restricted [68, 69], thus allowing the short-term adjustment of stromal NADPH redox state in response to changing environmental conditions [70]. The observed down-representation of ketol-acid reductoisomerase, a key enzyme in branched-chain amino acid synthesis, is suggestive of the plant need of preserving the existing energy and of stimulating alternative catabolic pathways to generate it [71, 72].

Although oxidative stress occurs as a result of drought stress, the relative abundance of tomato chloroplast proteins associated with stress defence was not significantly affected. Nevertheless, an alteration of proteins indirectly involved in cell defence was observed. In this context, noteworthy is the over-representation of cysteine synthase, a key enzyme directly involved in sulphur absorption and synthesis of cysteine, but also indirectly regulating the formation of methionine, glutathione, and sulphurated secondary metabolites [73, 74]. A protective effect can also be attributed to the over-representation of the plastid lipid-associated protein CHRC, which prevents oxidation of thylakoid membranes, and the down-representation of various polyphenol oxidases (e.g., PPO E and F) involved in degrading antioxidant polyphenols. A similar quantitative trend for these enzymes was observed in the drought-tolerant species *Craterostigma plantagineum* [75] and various tomato cultivars [76]. Our results on these proteins suggest a drought-tolerant character also for the tomato genotype here investigated. The latter authors also observed that PPO F-overexpressing plants suffer a greater chlorophyll bleaching than the wild-type counterpart, thus hypothesizing a possible loss of chlorophyll due to quinone production. This observation was in good agreement with our determination of the concomitant down-representation of PPOs and the constant content of chlorophylls in tomato plants after drought stress.

Despite the large number of reports on the response of crops to drought, poor information is currently available on plant recovery phase [23]. Following the rewatering cycle, tomato plants seemed to have a complete recovery, as demonstrated by physiological parameters and levels of metabolites returned to control values (Figs. 1d and 2). On the other hand, proteomic results from DR plants showed an evident adjustment of the corresponding protein repertoire, which was even larger than that measured for D counterparts (Fig. 4 and Table 1). Recovery after drought stress is a dynamic process that involves the rearrangement of many metabolic pathways to repair water depletion-induced damages and to resume plant growth [54]. The observed down-regulation of proteins involved in energy production and carbon metabolism such as components of both photosystems (e.g., plastocyanin, chlorophyll a-b binding protein4, etc.), Calvin-Benson cycle enzymes (e.g., phosphoglycerate kinase, phosphoribulokinase, etc.) and ATP synthase complex constituents (e.g., ATP β, γ, etc.) suggested a still ongoing reduction of plant metabolism following the severe stress and the short-term recovery applied. In contrast, evidences for a metabolism reactivation derived from the regulation of proteins involved in carbon/nitrogen ratio balancing, such as the increase of aspartate aminotransferase and the down-representation of PII-like protein [77], suggests the plant need for carbon sources to be redirected toward the Krebs cycle, presumably to cope with the above-mentioned reduction of enzymes involved in energy production. Further, the down-representation of glutamine synthetase, which assists the assimilation of NH_3_ generated through the photorespiration [78], reflects the increase of photosynthetic efficiency due to CO_2_ availability as revealed by stomatal conductance measurements (Fig. 1b). Coherent with a metabolism reactivation was also the reduction of the chloroplast defence proteins thioredoxin M4 and Cu-Zn superoxide dismutase, which are probably no more necessary to face ROS imbalance. Moreover, the accumulation of glutamate 1-semialdehyde 2,1-aminomutase and chlorophyll reductase NOL, which are both implicated in chlorophyll biosynthesis/degradation, indicated a reactivation of the photosynthetic pigment turnover. Finally, the down-representation of ISPH protein that participates in the non-mevalonate pathway controlling isoprene, phytol, carotenoid and plant hormone formation, might be related to the observed concomitant reduction of the ABA content [79].

To evaluate a putative transcriptional regulation of the genes associated with the proteins already identified as differentially represented in drought and recovery phases in tomato, we investigated the corresponding transcript accumulation. Some discrepancies were observed between transcript levels of the analysed genes and protein quantitative data and, in accordance with previous studies on other eukaryotes [80], they could be associated with translational regulation, post-translational modification or protein turnover events. Nevertheless, our qRT-PCR results were in good agreement with transcriptomic data from drought stressed *Arabidopsis* and tomato plants [23, 81, 82].

**Fig. 7:**
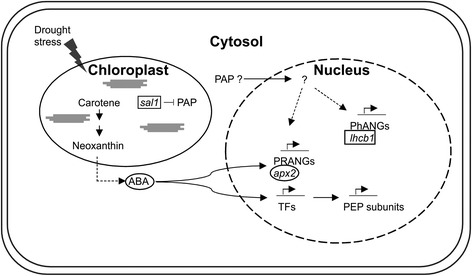
Model for retrograde and hormonal signaling pathways based on the results of this work. *Circles* and *squares* indicate the increase and the decrease of transcript levels and metabolites, respectively. *PhANGs* photosynthesis-associated nuclear genes, *PRANGs* plastid redox-associated nuclear genes, *ABA* abscisic acid, *apx2* cytosolic ascorbate peroxidase 2, *lhcb1* light harvesting complex b1, *sal1* 3′(2′),5′-bisphosphate nucleotidase, *PAP* 3′-phosphoadenosine 5′-phosphate

Chloroplast homeostasis and its subsequent readjustment in response to environmental stimuli requires a complex communication from organelle to nucleus, which involves one or more chloroplast signals that can interact with hormonal signaling network as demonstrated in *A. thaliana* mutants [83–85]. In this study, we demonstrated that also in an important crop such as tomato the chloroplast acts as an environmental sensor and organelle homeostasis is modified in response to drought. In particular, proteomic results suggested that the altered representation of several photosynthesis-associated nuclear-encoded plastid proteins may be due to a retrograde control. Both western blotting and qRT-PCR results on the light harvesting complex protein (gene), a favoured marker of retrograde signaling that is generally repressed under stress conditions [45, 46], confirmed our hypothesis as reported by literature. The up-regulation of ascorbate peroxidase 2 (*apx2*) and the down-regulation of *lhcb1* and *sal1*, encoding an enzyme that degrades 3′-phosphoadenosine 5′-phosphate (PAP) to adenosine monophosphate (AMP), and the 5-fold increase of ABA in drought-stressed tomato plants suggest the activation of both retrograde and hormonal crosstalks. As recently reviewed by Chan et al. [85] these processes are probably mediated by PAP (Fig. 7), although still unknown is the intersecting point between retrograde and hormone signaling in the stress response. It has been demonstrated that PAP regulates the expression of stress-induced genes through the attenuation of RNA catabolism mediated by 5′-3′ exoribonucleases (XRNs) [47], whereas no changes in relative abundance of *xrn2* and *xrn3* transcripts were observed in our drought-stressed compared to control plants. Similar results were reported in microarray data in drought-stressed *A. thaliana* plants [81, 82].

Increased ABA levels might affect the transcript levels of most chloroplast genes, including those encoding the subunits of Plastid-Encoded RNA Polymerase (PEP) as recently demonstrated by Yamburenko et al. [86]. They reported that the signal is transduced to the nucleus by group-A protein phosphatase 2c proteins and subclass III sucrose non-fermenting protein-related kinase 2, altering the activity of the transcription factors (e.g., *abi4*) and, as result, of many nuclear genes. This ultimately leads to the activation of the expression of relA-spotT-homolog (*rsh*) genes, coding for chloroplastic enzymes involved in the synthesis of guanosine-3′-5′bisdiphosphate (ppGpp), and of chloroplast transcription sigma factor 5 (*sig5*), supporting the core of PEP RNA polymerase to bind to promoter sequences. However, it is not clear whether this activation is a direct effect of ABA. Although a reduction of transcript accumulation of genes encoding subunits of PEP, except *rpoB*, was observed in our tomato plants, similarly to what already detected in *A. thaliana* [81, 82], this regulation did not seem associated with an up-regulation of both *rsh* (*rsh2* and *rsh3*) and *sig5* genes, in our plants. Thus, our results support the conclusion of Yamburenko et al. [87] on the need of further investigations in order to obtain a complete picture of the ABA effects on chloroplast gene expression.

## Conclusions

Chloroplast acts as environmental sensor by both coordinating the expression of nuclear-encoded plastid-localised proteins and mediating plant stress response. Our results effectively provide an original overview on the chloroplast response to long-term drought stress and subsequent recovery in tomato. Photosynthesis was confirmed as the main process affected by water deficit in association with energy-related functional species, and peculiar traits of tolerance were identified, which can be relevant targets to improve the response to drought. Evidence for metabolism reactivation was obtained following the rewatering cycle by metabolite and physiological data, while proteomics revealed a still ongoing adjustment of the chloroplast protein repertoire. In addition to unveil putative effectors and mechanisms involved in chloroplast proteome adjustment in response to water withholding, this investigation originally describes in tomato the expression of genes already known to be involved in different chloroplast-to-nucleus signaling pathways in *A. thaliana* mutants. Although our results suggest the activation of a specific retrograde signaling pathway and interconnection with ABA signaling network in tomato, the involvement of such pathway, and its fine regulation as a function of the drought intensity need to be further investigated through the development and characterization of ad hoc designed plant mutants.

## Additional files

Additional file 1: Table S1. Nucleotide sequence of primers used for qRT-PCR analyses. **Figure S1.** Representative 2D-DIGE showing the resolution of about 2600 protein spots from tomato chloroplasts in IEF using pH 3-10 NL 18 cm strips, followed by 12% T SDS-PAGE. Spot visualization was obtained with a Typhoon fluorescence scanner. Differentially represented spots further subjected to nLC-ESI-LIT-MS/MS analysis are highlighted. A Cy-2 labeled pooled sample mixture was used as an internal standard for quantitative measurements. **Table S2.** Listed are data referring to spot number, NCBI protein accession number, protein description, MASCOT score value, theoretical and experimental molecular mass and pI values, total and unique peptides detected, sequence coverage (%), EMPAI score value, fold change in drought stressed plants with respect to control, and fold change in recovered drought stressed plants with respect to well-watered control of the same age. In addition, data referring to protein spots not showing significant quantitative variations are reported in italics and are highlighted in green. Protein species showing either an incoherent quantitative trend or a constant trend among the experimental conditions are highlighted in blue. (PDF 2377 kb)
Additional file 2: Table S3. Identification details for the proteins observed as differentially represented by 2D-DIGE. Reported are spot number, protein accession/description, MASCOT score, total and unique peptides detected, identification rank and unicity, experimental and theoretical peptide mass value, peptide charge state, peptide mass error, number of peptide missed cleavage(s), peptide score, peptide expectation value and sequence, including amino acid(s) modification. (XLSX 1109 kb)

## Abbreviations

ABA: Abscisic acid
AMP: Adenosine monophosphate
*apx2*: Ascorbate peroxidase 2
Apx-TL29: Ascorbate peroxidase
ATPB: ATP synthase β
CSP41b: Chloroplast stem-loop binding protein 41 kDa
FKBP13: Peptidyl-prolyl *cis-trans* isomerase
FNR: Ferredoxin-NADP reductase
GS2: Glutamine synthase
Lhcb1: Light harvesting complex protein
MDH: Malate dehydrogenase
OEC1: 33 kDa precursor protein of oxygen-evolving complex
OEE1: Oxygen-evolving enhancer protein 1
PAP: 3′-phosphoadenosine 5′-phosphate
PEP: Plastid-encoded RNA polymerase
ppGpp: Guanosine-3′-5′bisdiphosphate
PPO E: Polyphenol oxidase E
PPO F: Polyphenol oxidase F
PRK: Phosphoribulokinase
Pro: Proline
PsbQ: Photosystem II oxygen-evolving complex protein 3
RA: Ribulose-1,5-bisphosphate carboxylase/oxygenase activase
RbcL: Ribulose-1,5-bisphosphate carboxylase/oxygenase large subunit
*sal1*: Gene encodes a bifunctional phosphatase protein
SBPase: Chloroplast sedoheptulose-1,7-bisphosphatase
TK: Transketolase
XRNs: Exoribonucleases
