# Supplementary material for: Chloroplast proteome response to drought stress and recovery in tomato (Solanum lycopersicum L.)
Source: BMC Plant Biol. 2017 Feb 10;17:40. doi: 10.1186/s12870-017-0971-0 (PMC5301458; doi:10.1186/s12870-017-0971-0)
Supplement: Additional file 1: Table S1. — Nucleotide sequence of primers used for qRT-PCR analyses. Figure S1. Representative 2D-DIGE showing the resolution of about 2600 protein spots from tomato chloroplasts in IEF using pH 3-10 NL 18 cm strips, followed by 12% T SDS-PAGE. Spot visualization was obtained with a Typhoon fluorescence scanner. Differentially represented spots further subjected to nLC-ESI-LIT-MS/MS analysis are highlighted. A Cy-2 labeled pooled sample mixture was used as an internal standard for quantitative measurements. Table S2. Listed are data referring to spot number, NCBI protein accession number, protein description, MASCOT score value, theoretical and experimental molecular mass and pI values, total and unique peptides detected, sequence coverage (%), EMPAI score value, fold change in drought stressed plants with respect to control, and fold change in recovered drought stressed plants with respect to well-watered control of the same age. In addition, data referring to protein spots not showing significant quantitative variations are reported in italics and are highlighted in green. Protein species showing either an incoherent quantitative trend or a constant trend among the experimental conditions are highlighted in blue. (PDF 2377 kb) [file 12870_2017_971_MOESM1_ESM.pdf]

Table S1. Nucleotide sequence of primers used for qRT-PCR analyses.

Gene[a]	Forward (5'→3')	Reverse (5'→3')	References
abi4	GTGGTAGATCCTATGACTGTTGAC	CGACAAGTTCAGAATCCAAGTC	[1, 2]
abp19a-like	CAGGTGTCAATGGGCTTGGA	TAAAGCACCTCCGATGCTCC	This work
apx2	TCAGGATTTGAGGGAGCATGGA	TTTGTCTGATGGTAGCTGGAGGAG	[3]
apx-tl29	CCTGATCCAGAGGGAAGGGT	TAACTGACGAGGGCCCAAAC	[4]
cpn60α	AAGGCATTAGTAGCTCCGGC	CCCATCTTCCATTTGCTGCC	This work
Cu-Zn sod	TTGGACGAGCACTTGTGGTT	CCAATCGTCCACCAGCATTG	This work
ef-1α	CTCCATTGGGTCGTTTTGCT	GGTCACCTTGGCACCAGTTG	Internal control
fc1	GGTCGTGTTCCTGCTCTGAATTG	GTGCCAGTGGACGTTGACATT	[5]
ftsh2	CGGTCTAACGGAGGAATGGG	ACACCAGTGTTTGGCTCCAT	[4]
ftsh-like	GCACTTGGTGGAAGAGTTGC	CCTTGCCACTCGTGAGACTT	[4]
glk2	GTACCAGGCACTCCTTGTTT	CAGAGGCAACTGTGTCAACT	[6]
gun1	CATGGACTTCTCATGGGGCA	TGCCACAGCATGTCAGTCAA	[7]
lea	CGAAGGAGAAGGCTAGTGGA	AACAAGTGAGGAGCATCGCT	[4]
lhcb1	ATGCTTGGTGCTCTTGGATG	TGCCCAAGTAGTCAAGTCCA	[8, 9]
por	AGGCTTCTTTTCCCACCATT	CAGTAGACACCTGATTTTGT	[4]
ppoF	AGAACCAGGCATGGGTACTA	ACCCATGTCTTCACCGTGTC	[4]
psbP6	CAAACAGTGGGCATCATCACAGA	GGCACTCACTCTATGTAAGGGAAGA	This work
psbQ	GCCAAGGAGATTGTTAGTGTCAAGGA	AAGGTCATAGCGAAGGTATTCTGC	This work
ptm	TCAGAGGTCACCAGAGAAACCA	CCCATACAGGAAGGAGAGTCAGTAA	[10]
rbcL	CTGCGAATCCCTCCTGCTTA	CCAACAGGGGACGACCATAC	This work
rpoA	TAACTCCGAAAGAAGCACTT	AGGAACCGTGTGTTGATTGTCC	[11]
rpoB	TGTATCCGCGGGATTAATTTGGA	ATCAAGGTTCAGTTTTCGGTTCA	[11]
rpoC1	AGAGCCGATTGTATGGGAAA	GCTGGAATGCCTGTATGCCTAATC	[11]
rpoC2	GAGGGTTGAGGGTTGGAACAAA	TTAACCAAAGAGATTCGGCT	[11]
rsh2	AGCACAATGAACTGTCATCC	ATAAGAGACAGATCCATCAC	[12]
rsh3	GACCGTGAAGGATCTGTTGG	CTAACTGGTTCGTGGTTCAA	[12]
sal1	ATGGTGCTTTGTCCCGTGGAGATG	GCAGCATCTGAGACAACACCT	[3]
sig5	GCTCCAGAGGATACCAACAGATGA	GGCGTGGAGAGAGAAGATAGGTTTAG	[13]
why1	AAGGCAGAGTTCGCAGTTCTTG	GTTTGAGCGACTGGCGTCTT	[14, 15]
xrn2	CACAGTAGAGATTATCGTGGTC	GTGCTGAGGATGACTGGCGTA	[3]
xrn3	GTGTATGGGCTCCGCAAGTAAA	GCCCTCTATCGTTTCTTCTTCCTA	[3]
zat10	GCGTTCATAGTGGAAGCGTTGT	AGGCAGGCATGTTTAGGTCAAAG	[3]

[a] *abi4*: abscisic acid insensitive4; *abp19a-like*: auxin binding protein; *apx-tl29*: ascorbate peroxidase (TL29); *apx2*: cytosolic ascorbate peroxidase; *cpn60α*: Rubisco large subunit-binding protein subunit alpha; *Cu-Zn sod*: superoxide dismutase; *fc1*: ferrochelatase1; *ftsH2* and *ftsH-like*: ATP-dependent zinc metalloprotease FtsH2 and FtsH-like; *glk2*: golden 2-like2; *gun1*: genome uncoupled 1; *lea*: late embryogenesis abundant; *lhcb1*: light harvesting complex b1; *por*: protochlorophyllide reductase; *ppoF*: polyphenol oxidase F; *psbP*: psbP domain-containing protein 6; *psbQ*: photosystem II oxygen-evolving complex protein 3; *ptm*: chloroplast envelope-bound plant homeodomain; *rbcL*: ribulose-1,5-bisphosphate carboxylase/oxygenase large subunit; *rpoA*, *rpoB*, *rpoC1*, *rpoC2:* subunits of Plastid-Encoded RNA Polymerase; *rsh2*, *rsh3*: relA/spoT homolog 2; *sal1*: 3'(2'),5'-bisphosphate nucleotidase; *sig5*: sigma factor 5; *why1*: whirly; *xrn2*, *xrn3:* 5'-3' exoribonuclease 2 and 3; *zat10*: C2H2 zinc finger transcription factor ZAT10.

References

1. Zhang ZW, Feng LY, Cheng J, Tang H, Xu F, Zhu F, Zhao ZY, Yuan M, Chen YE, Wang JH et al. The roles of two transcription factors, ABI4 and CBFA, in ABA and plastid signalling and stress responses. Plant Mol Biol. 2013; 83(4-5):445-458.
2. León P, Gregorio J, Cordoba E. ABI4 and its role in chloroplast retrograde communication. Front Plant Sci. 2012; 3:304.
3. Estavillo GM, Crisp PA, Pornsiriwong W, Wirtz M, Collinge D, Carrie C, Giraud E, Whelan J, David P, Javot H et al. Evidence for a SAL1-PAP chloroplast retrograde pathway that functions in drought and high light signaling in Arabidopsis. Plant Cell 2011; 23(11):3992-4012.
4. Iovieno P, Punzo P, Guida G, Mistretta C, Van Oosten MJ, Nurcato R, Bostan H, Colantuono C, Costa A, Bagnaresi P et al. Transcriptomic changes drive physiological responses to progressive drought stress and rehydration in tomato. Front Plant Sci. 2016; 7:371.
5. Woodson JD, Perez-Ruiz JM, Chory J. Heme synthesis by plastid ferrochelatase I regulates nuclear gene expression in plants. Curr Biol. 2011; 21(10):897-903.
6. Waters MT, Wang P, Korkaric M, Capper RG, Saunders NJ, Langdale JA. GLK transcription factors coordinate expression of the photosynthetic apparatus in Arabidopsis. Plant Cell 2009; 21(4):1109-1128.

7. Koussevitzky S, Nott A, Mockler TC, Hong F, Sachetto-Martins G, Surpin M, Lim IJ, Mittler R, Chory J. Signals from chloroplasts converge to regulate nuclear gene expression. Science 2007; 316(5825):715-719.

8. Tiller N, Bock R. The translational apparatus of plastids and its role in plant development. *Molecular Plant* 2014, 7(7):1105-1120.

9. Zhang ZW, Zhang GC, Zhu F, Zhang DW, Yuan S. The roles of tetrapyrroles in plastid retrograde signaling and tolerance to environmental stresses. Planta 2015; 242(6):1263-1276.

10. Sun X, Feng P, Lu C, Xu X, Zhang L. A chloroplast envelope-bound PHD transcription factor mediates chloroplast signals to the nucleus. Nat Comm. 2011; 2:477.

11. Yamburenko MV, Zubo YO, Vankova R, Kusnetsov VV, Kulaeva ON, Borner T. Abscisic acid represses the transcription of chloroplast genes. J Exp Bot. 2013; 64(14):4491-4502.

12. Yamburenko MV, Zubo YO, Borner T. Abscisic acid affects transcription of chloroplast genes via protein phosphatase 2C-dependent activation of nuclear genes: repression by guanosine-3-5-bisdiphosphate and activation by sigma factor 5. Plant J. 2015; 82(6):1030-1041.

13. Woodson JD, Perez-Ruiz JM, Schmitz RJ, Ecker JR, Chory J. Sigma factor-mediated plastid retrograde signals control nuclear gene expression. Plant J. 2013; 73(1):1-13.

14. Foyer CH, Karpinska B, Krupinska K. The functions of WHIRLY1 and REDOX-RESPONSIVE TRANSCRIPTION FACTOR 1 in cross tolerance responses in plants: a hypothesis. Philos Trans R Soc Lond B Biol Sci. 2014;369:20130226.

15. Isemer R, Krause K, Grabe N, Kitahata N, Asami T, Krupinska K. Plastid located WHIRLY1 enhances the responsiveness of Arabidopsis seedlings toward abscisic acid. Front Plant Sci. 2012; 3:283.

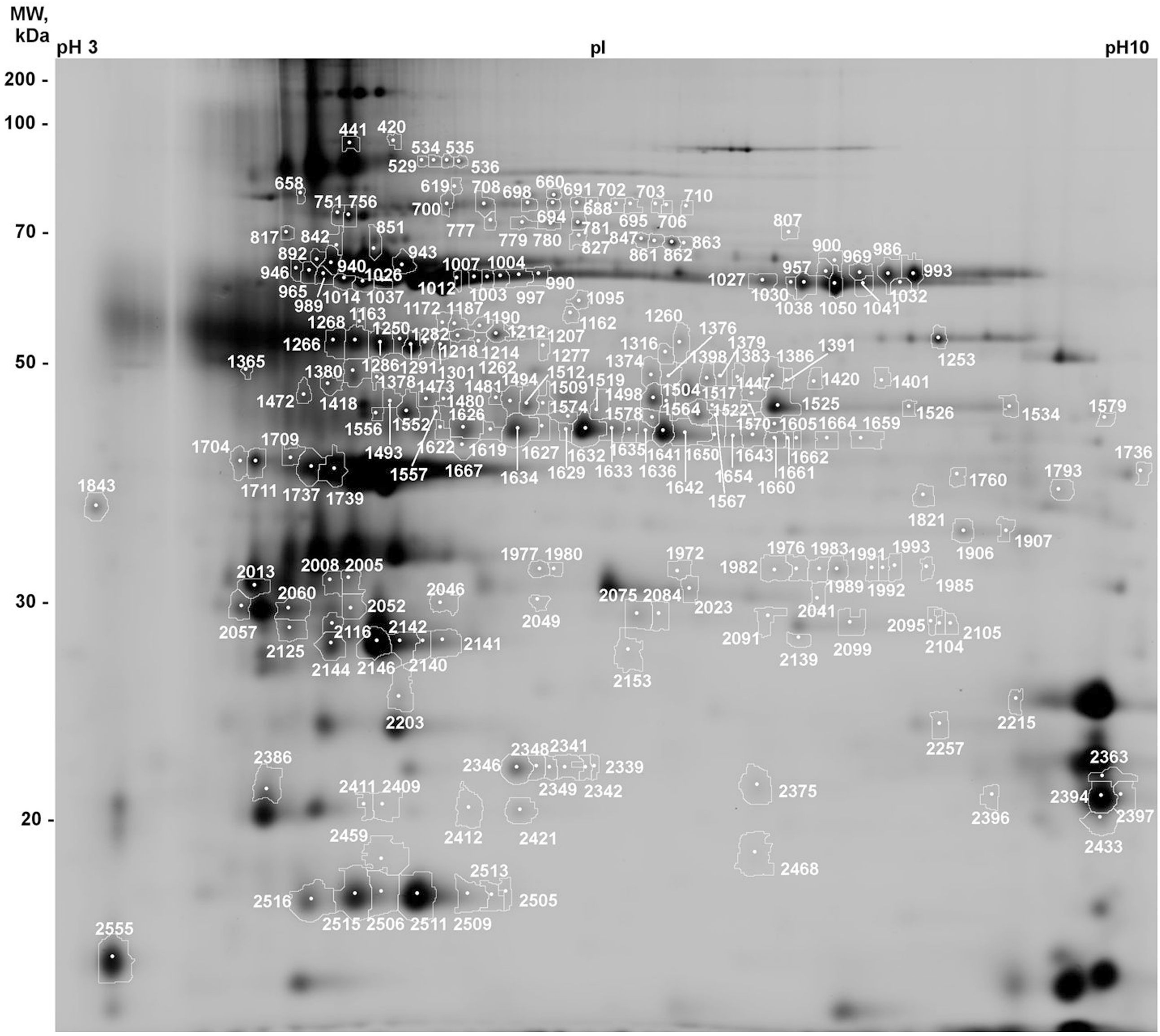

Fig. S1 Representative 2D-DIGE showing the resolution of about 2600 protein spots from tomato chloroplasts in IEF using pH 3-10 NL 18 cm strips, followed by 12% T SDS-PAGE. Spot visualization was obtained with a Typhoon fluorescence scanner. Differentially represented spots further subjected to nLC-ESI-LIT-MS/MS analysis are highlighted. A Cy-2 labeled pooled sample mixture was used as an internal standard for quantitative measurements.

Table S2. Listed are data referring to spot number, NCBI protein accession number, protein description, MASCOT score value, theoretical and experimental molecular mass and pI values, total and unique peptides detected, sequence coverage (%), EMPAI score value, fold change in drought stressed plants with respect to control, and fold change in recovered drought stressed plants with respect to well-watered control of the same age. In addition, data referring to protein spots not showing significant quantitative variations are reported in italics and highlighted in green. Proteins showing either an incoherent quantitative trend or a constant trend among the experimental conditions are highlighted in blue.

												Drought Vs Cntr		Drought Rw Vs Cntr Rw	
Spot	NCBI code	Description	Mascot Score	$Mass_{th}$	$Mass_{ex}$	pI_{th}	pI_{ex}	Peptides	Unique Peptides	Coverage %	emPAI score	Ratio	T-test	Ratio	T-test
420	*460400133*	*Aminopeptidase N-Like*	*695*	*102406*	*102406*	*5.55*	*5.90*	*13*	*13*	*18.7*	*0.59*				
441	*460400133*	*Aminopeptidase N-Like*	*100*	*102406*	*78939*	*5.55*	*5.68*	*2*	*2*	*3*	*0.07*				
529	170435	Atp-Dependent Protease (Cd4B)	1435	102463	76249	5.86	6.05	30	28	36.4	1.51			-1.97	0.003
534	170435	Atp-Dependent Protease (Cd4B)	1813	102463	76249	5.86	6.11	40	31	34.2	2.58			-1.78	0.019
535	170435	Atp-Dependent Protease (Cd4B)	1970	102463	76249	5.86	6.18	66	37	41	3.43			-2.18	0.017
536	170435	Atp-Dependent Protease (Cd4B)	1759	102463	76166	5.86	6.24	70	32	36.3	2.46			-1.99	0.0031
619	*27462474*	*GcpE*	*662*	*82805*	*72384*	*5.76*	*6.22*	*14*	*14*	*20.4*	*0.77*				
658	460369188	Stromal 70 Kda Heat Shock-Related Protein	1098	74965	71449	5.20	5.43	28	21	30.4	1.17	-1.75	0.021		
	158635118	Heat Shock Protein 70	473	74415	71449	5.41	5.43	13	10	15.9	0.48				
660	460406209	Transketolase	1525	80268	71217	5.97	6.72	106	31	53.8	3.24			-2.03	0.034
698	460406209	Transketolase	924	80268	70068	5.97	6.59	27	17	25.1	1.06			2.96	0.015
688	37538489	Ftsh-Like Protein Precursor	977	76138	70220	5.78	6.91	15	15	31.9	0.95			3.37	0.021
691	37538489	Ftsh-Like Protein Precursor	869	76138	70144	5.78	6.84	12	12	24.2	0.77			2.73	0.037
694	37538489	Ftsh-Like Protein Precursor	399	76138	70068	5.78	6.72	6	5	10.9	0.27			3.11	0.032
695	37538489	Ftsh-Like Protein Precursor	765	76138	69992	5.78	7.11	12	11	25.1	0.69			2.86	0.0028
700	37538489	Ftsh-Like Protein Precursor	1069	76138	69992	5.78	6.18	20	16	32.3	1.35			2.48	0.0043
702	2150027	Nadp-Malic Enzyme	417	70598	70598	5.93	7.01	9	8	15	0.51			2.16	0.032
708	37538489	Ftsh-Like Protein Precursor	740	76138	69917	5.78	6.37	17	13	22.7	0.95			2.46	0.0021
703	37538489	Ftsh-Like Protein Precursor	686	76138	69992	5.78	7.24	11	11	23.2	0.69			3.37	0.0052
706	37538489	Ftsh-Like Protein Precursor	392	76138	69765	5.78	7.30	6	6	12.6	0.33			3.56	0.014
710	37538489	Ftsh-Like Protein Precursor	283	76138	69690	5.78	7.39	4	4	8.6	0.21			2.48	0.0022
751	460395390	Atp-Dependent Zinc Metalloprotease Ftsh 2	2136	74424	68715	6.00	5.62	50	39	56.6	8.38			-1.92	0.052
756	460395390	Atp-Dependent Zinc Metalloprotease Ftsh 2	1802	74424	68492	6.00	5.68	83	31	42.7	3.75			-1.94	0.047
777	*460369951*	*Ferredoxin-Nitrite Reductase*	*475*	*66478*	*67680*	*5.84*	*6.40*	*10*	*9*	*18.1*	*0.63*				
779	*460369951*	*Ferredoxin-Nitrite Reductase*	*602*	*66478*	*67387*	*5.84*	*6.56*	*14*	*13*	*21.3*	*0.82*				
780	*460369951*	*Ferredoxin-Nitrite Reductase*	*515*	*66478*	*67241*	*5.84*	*6.71*	*12*	*10*	*19.8*	*0.54*				
781	460401031	Polyphenol Oxidase F	604	66950	67387	6.04	6.85	15	13	21.5	1.13	-2.75	0.055		
817	460411525	Rubisco Large Subunit-Binding Protein Subunit Alpha	1974	62032	65728	5.21	5.21	50	33	61.4	8.13			-1.74	0.022
842	460379814	Rubisco Large Subunit-Binding Protein Subunit Beta	1348	64527	64459	5.46	5.62	23	21	44	2.24	-1.77	0.0045		
	460411525	Rubisco Large Subunit-Binding Protein Subunit Alpha	644	62032	64459	5.21	5.62	11	10	23.5	0.9				
851	460366131	Rubisco Large Subunit-Binding Protein Subunit Beta	1890	63238	64041	5.72	5.81	58	33	64.4	5.58	-1.52	0.063	-1.7	0.021
827	*260528216*	*Inosine Monophosphate Cyclohydrolase*	*654*	*66199*	*65728*	*6.21*	*6.85*	*18*	*15*	*28.2*	*1.15*				
847	22733	Polyphenol Oxidase Precursor	353	66822	65232	6.36	7.17	8	7	11.9	0.46	-1.66	0.024		
861	22733	Polyphenol Oxidase Precursor	427	66822	65020	6.36	7.23	11	9	18.4	0.54	-1.62	0.016		
	460395681	Ketol-Acid Reductoisomerase	386	64192	65020	6.31	7.23	8	7	15	0.57				
862	460401033	Polyphenol Oxidase E	801	66808	64809	6.36	7.32	28	16	32.7	1.38	-1.9	0.057		
863	460395681	Ketol-Acid Reductoisomerase	282	64192	64739	6.31	7.39	6	6	14.3	0.4	-1.86	0.029		
892	84371968	Atp Synthase Cf1 Alpha Subunit	938	55434	62668	5.14	5.52	77	18	35.7	2.23	-1.85	0.014		
940	84371968	Atp Synthase Cf1 Alpha Subunit	1073	55434	62194	5.14	5.59	73	21	40.6	3.47	-1.7	0.015		
943	84371968	Atp Synthase Cf1 Alpha Subunit	970	55434	61992	5.14	5.95	103	18	36.9	3.77	-1.91	0.054		
946	84371990	Atp Synthase Cf1 Beta Subunit	1320	53491	61524	5.28	5.41	58	25	63.1	6.06	-2.06	0.016		
965	84371990	Atp Synthase Cf1 Beta Subunit	1173	53421	61258	5.14	5.48	46	21	57	5.19	-2.28	0.00043		
989	84371990	Atp Synthase Cf1 Beta Subunit	1174	53421	60861	5.14	5.55	54	20	58.6	4.41	-1.89	0.014		
1014	84371990	Atp Synthase Cf1 Beta Subunit	1476	53491	60335	5.28	5.66	108	24	58.6	7.64	-1.95	0.0086		
	460386362	Atp Synthase Subunit Beta	1066	59684	60335	5.74	5.66	40	19	47.8	2.35				
1026	84371990	Atp Synthase Cf1 Beta Subunit	1660	53491	60074	5.28	5.85	186	29	67.5	13.8	-1.74	0.032		
1037	84371990	Atp Synthase Cf1 Beta Subunit	1220	53491	59944	5.28	5.75	72	22	59.2	5.17	-1.71	0.011		
990	84371990	Atp Synthase Cf1 Beta Subunit	1859	53491	60927	5.28	6.65	154	31	80.7	19.73			-1.75	0.018
997	84371990	Atp Synthase Cf1 Beta Subunit	1612	53491	60729	5.28	6.54	91	26	66.1	12.84			-1.77	0.0063
1003	84371990	Atp Synthase Cf1 Beta Subunit	1510	53491	60532	5.28	6.38	158	25	72.1	11.94			-1.82	0.016
1004	84371990	Atp Synthase Cf1 Beta Subunit	1798	53491	60597	5.28	6.45	217	29	70.3	26.15			-1.81	0.011
1007	84371990	Atp Synthase Cf1 Beta Subunit	1525	53491	60532	5.28	6.32	161	23	66.1	17.12			-2.32	0.024
1012	84371990	Atp Synthase Cf1 Beta Subunit	2070	53491	60335	5.28	6.23	219	33	83.1	39.67			-2.67	0.058
1207	84371990	Atp Synthase Cf1 Beta Subunit	1104	53491	54021	5.28	6.65	48	21	67.9	4.04			-2.65	0.024
807	84371991	Ribulose-1.5-Bisphosphate Carboxylase/Oxygenase Large Subunit	163	53434	66086	6.55	7.92	5	4	8.8	0.31			1.58	0.047
900	*84371991*	*Ribulose-1.5-Bisphosphate Carboxylase/Oxygenase Large Subunit*	*1009*	*53434*	*62464*	*6.55*	*8.15*	*57*	*19*	*37.5*	*3.72*				
993	84371991	Ribulose-1.5-Bisphosphate Carboxylase/Oxygenase Large Subunit	1059	53434	60927	6.55	8.55	52	20	38.6	5.62			-1.66	0.038
969	*84371991*	*Ribulose-1.5-Bisphosphate Carboxylase/Oxygenase Large Subunit*	*790*	*53434*	*60993*	*6.55*	*8.28*	*25*	*14*	*29.6*	*2.37*				
986	*349144*	*Ribulose-1.5-Bisphosphate Carboxylase/Oxygenase Large Subunit*	*947*	*53398*	*60927*	*6.55*	*8.43*	*51*	*18*	*35*	*3.13*				
957	*349144*	*Ribulose-1.5-Bisphosphate Carboxylase/Oxygenase Large Subunit*	*1334*	*53398*	*61191*	*6.55*	*8.11*	*95*	*27*	*49.1*	*12.01*				

Spot	NCBI code	Description	Mascot Score	$Mass_{th}$	$Mass_{ex}$	pI_{th}	pI_{ex}	Peptides	Unique Peptides	Coverage %	emPAI score	Drought Vs Cntr Ratio	Drought Vs Cntr T-test	Drought Rw Vs Cntr Rw Ratio	Drought Rw Vs Cntr Rw T-test
1027	84371991	Ribulose-1.5-Bisphosphate Carboxylase/Oxygenase Large Subunit	896	53434	60074	6.55	7.79	42	17	31.7	2.86			-2.56	0.039
1030	84371991	Ribulose-1.5-Bisphosphate Carboxylase/Oxygenase Large Subunit	1110	53434	59815	6.55	7.93	43	21	37.5	5.62			-2.25	0.0074
1032	349144	Ribulose-1.5-Bisphosphate Carboxylase/Oxygenase Large Subunit	1414	53398	59815	6.55	8.48	94	28	50.9	12.91			-1.88	0.016
1038	349144	Ribulose-1.5-Bisphosphate Carboxylase/Oxygenase Large Subunit	1405	53398	59879	6.55	8.00	100	29	53.5	9.62			-3.79	0.022
1041	84371991	Ribulose-1.5-Bisphosphate Carboxylase/Oxygenase Large Subunit	1386	53434	59750	6.55	8.30	75	28	48.4	7.67			-2.13	0.0066
1050	349144	Ribulose-1.5-Bisphosphate Carboxylase/Oxygenase Large Subunit	1938	53398	59685	6.55	8.15	178	36	65.8	28.24			-3.69	0.0075
1095	460376468	Atp Sulfurylase 1	378	52376	57713	6.94	6.85	13	10	23.6	0.86			-3.06	0.0091
1162	642911	Glutamate 1-Semialdehyde 2.1-Aminomutase	702	51722	56292	6.54	6.81	16	13	30.6	1.65			1.51	0.037
1163	262176919	Isph Protein	459	52147	55385	5.38	5.74	11	11	23.2	1			-1.62	0.02
1172	460391817	Elongation Factor Tua	771	56286	55205	6.69	6.16	28	14	35.5	1.97			-6.22	0.023
1187	460391817	Elongation Factor Tua	815	56286	55145	6.69	6.22	21	15	30.3	2.84			-2.89	0.013
1190	460391817	Elongation Factor Tua	1246	56286	54907	6.69	6.34	64	22	48.6	7.84			-3.18	0.026
1212	460396820	Phosphoglycerate Kinase	1325	50592	54080	7.66	6.53	61	23	61.8	5.36			-2.56	0.021
1214	460396820	Phosphoglycerate Kinase	1499	50592	54021	7.66	6.43	85	27	68.7	8.75			-3.48	0.02
1218	460396820	Phosphoglycerate Kinase	1071	50592	53846	7.66	6.24	41	19	50.8	3.78			-2.59	0.0059
1250	460367196	Glutamine Synthetase	741	47852	53439	6.29	5.94	58	15	34.7	2.59			-5.09	0.0045
1260	460415552	Glyceraldehyde-3-Phosphate Dehydrogenase B	828	48539	53150	6.72	7.34	47	15	33.3	2.8			-2.47	0.037
1316	460385509	Glyceraldehyde-3-Phosphate Dehydrogenase B	878	51421	52067	6.86	7.29	59	17	36.3	3.06			-2.42	0.039
1401	460382243	Glyceraldehyde-3-Phosphate Dehydrogenase A	397	42940	49214	8.46	8.39	11	7	17	1.12			-3.35	0.0015
1420	460382243	Glyceraldehyde-3-Phosphate Dehydrogenase A	1033	42940	49107	8.46	8.05	54	16	44.1	7.09			-3.49	0.033
1253	460398807	Aspartate Aminotransferase	270	50984	50984	9.06	9.12	5	5	10.8	0.33			1.49	0.0063
1266	460401823	Ribulose Bisphosphate Carboxylase/Oxygenase Activase 1	681	49054	53381	8.15	5.60	16	14	36.3	1.24			-3.37	0.0044
1268	460401823	Ribulose Bisphosphate Carboxylase/Oxygenase Activase 1	843	49054	53323	8.15	5.71	23	17	35.1	2.23			-3.89	0.0068
1262	460401823	Ribulose Bisphosphate Carboxylase/Oxygenase Activase 1	1150	49054	53266	8.15	6.34	37	24	51	3.03			-2.81	0.0082
1277	460401823	Ribulose Bisphosphate Carboxylase/Oxygenase Activase 1	921	49054	52806	8.15	6.67	16	16	38.1	2.01			-2.39	0.041
1282	460401823	Ribulose Bisphosphate Carboxylase/Oxygenase Activase 1	988	49054	53150	8.15	6.07	84	15	35.4	4.82			-4.07	0.036
1286	460401823	Ribulose Bisphosphate Carboxylase/Oxygenase Activase 1	1297	49054	53150	8.15	5.84	98	25	42.9	8.03			-4.7	0.0016
1291	460401823	Ribulose Bisphosphate Carboxylase/Oxygenase Activase 1	1161	49054	52863	8.15	6.00	91	21	37.6	9.46			-4.17	0.0094
1301	460401823	Ribulose Bisphosphate Carboxylase/Oxygenase Activase 1	1398	49054	52920	8.15	6.15	60	25	48.1	5.74			-3.5	0.0031
1374	26453355	Mrna Binding Protein Precursor	485	44084	49804	7.1	7.22	11	9	30.2	1.26			-2.17	0.012
1376	26453355	Mrna Binding Protein Precursor	575	44084	49643	7.1	7.31	11	10	32.9	1.26			-1.79	0.0089
1398	26453355	Mrna Binding Protein Precursor	619	44084	49428	7.1	7.50	20	13	34.4	1.66			-2.94	0.0021
1365	38604456	Fructokinase 3	895	41803	50292	5.57	5.16	16	16	51.8	2.05	1.57	0.058		
	460398434	Cysteine Synthase	491	41255	50292	5.41	5.16	9	8	26.4	1.19				
1380	460400830	Phosphoribulokinase	996	44793	50183	5.96	5.70	66	18	62.3	3.97			-2.12	0.019
1378	*460400830*	*Phosphoribulokinase*	*643*	*44793*	*49589*	*5.96*	*5.82*	*18*	*13*	*31.8*	*1.83*				
	238563983	*Chloroplast Sedoheptulose-1.7-Bisphosphatase*	*463*	*43017*	*49589*	*6.07*	*5.82*	*10*	*9*	*24.6*	*1.3*				
1418	460400830	Phosphoribulokinase	603	44793	48948	5.96	5.57	16	13	33.3	1.62			-2.19	0.00067
	238563983	Chloroplast Sedoheptulose-1.7-Bisphosphatase	519	43017	48948	6.07	5.57	36	11	28.4	2.8				
1472	238563983	Chloroplast Sedoheptulose-1.7-Bisphosphatase	954	43017	47899	6.07	5.45	95	20	44.2	6.4			-2.1	0.015
1379	460391607	Chloroplast Stem-Loop Binding Protein Of 41 Kda B	674	42596	49643	7.67	7.57	15	13	32.7	1.75			-3.27	0.0018
1383	460391607	Chloroplast Stem-Loop Binding Protein Of 41 Kda B	541	42596	49535	7.67	7.66	15	11	31.9	1.75			-2.00	0.014
1386	460391607	Chloroplast Stem-Loop Binding Protein Of 41 Kda B	1210	42596	49643	7.67	7.83	43	24	60.4	9.58			-5.83	0.024
1391	460391607	Chloroplast Stem-Loop Binding Protein Of 41 Kda B	316	42596	49214	7.67	7.91	7	7	22.2	0.66			-2.64	0.015
1447	460391607	Chloroplast Stem-Loop Binding Protein Of 41 Kda B	432	42596	47950	7.67	7.73	9	9	23.2	0.96	-1.97	0.011		
1667	*460365514*	*Uncharacterized Protein Loc101254513*	*735*	*37384*	*43306*	*7.52*	*6.26*	*26*	*14*	*36.7*	*4.09*				
1473	460371306	Photosystem Ii Stability/Assembly Factor Hcf136	787	44248	47485	6.25	6.07	20	12	41.4	1.44			-2.29	0.016
1480	460371306	Photosystem Ii Stability/Assembly Factor Hcf136	794	44248	47434	6.25	6.16	20	11	36.2	2.11			-2.51	0.014
1481	460371306	Photosystem Ii Stability/Assembly Factor Hcf136	315	44248	47588	6.25	6.43	4	4	17.5	0.38			-1.88	0.0065
1493	460371306	Photosystem Ii Stability/Assembly Factor Hcf136	825	44248	47280	6.25	5.89	16	11	40.1	1.44			-1.88	0.000031
1494	460371306	Photosystem Ii Stability/Assembly Factor Hcf136	499	44248	47280	6.25	6.50	8	6	24.1	0.76			-2.45	0.0019
	460370090	Fructose-Bisphosphate Aldolase 2	445	43032	47280	6.85	6.50	12	9	22.9	0.79				
1517	460373820	Atp Synthase Gamma Chain	554	41752	46872	8.15	7.53	12	9	26.8	1.36			-2.17	0.048
	460373309	Fructose-Bisphosphate Aldolase 2	488	42656	46872	8.15	7.53	11	9	28.1	1.13				
1498	460373820	Atp Synthase Gamma Chain	828	41752	47537	8.15	7.23	46	13	42.4	5.63			-2.27	0.015
1504	460373820	Atp Synthase Gamma Chain	562	41752	47228	8.15	7.29	15	9	27.9	2.06			-2.12	0.031
1522	460373820	Atp Synthase Gamma Chain	828	41752	46821	8.15	7.72	31	15	39	2.33	-2.36	0.067		
1525	460373820	Atp Synthase Gamma Chain	1051	41752	46872	8.15	7.86	69	18	44.6	10.11			-2.48	0.0065
1526	460373820	Atp Synthase Gamma Chain	729	41752	46719	8.15	8.53	21	15	35.3	2.96			-2.34	0.02
1534	460373820	Atp Synthase Gamma Chain	785	41752	46719	8.15	9.28	38	14	39	4.58	-2.18	0.038		
1605	460373820	Atp Synthase Gamma Chain	580	41752	45176	8.15	7.85	14	9	28.6	1.58			-2.00	0.00038
1556	460375513	Fructose-Bisphosphate Aldolase 2	665	42872	46166	6.07	5.82	15	12	38.7	2.23			-2.14	0.034
1509	460370090	Fructose-Bisphosphate Aldolase 2	763	43032	47075	6.85	6.67	45	15	42.1	3.13			-4.45	0.015
1512	460370090	Fructose-Bisphosphate Aldolase 2	846	43032	47075	6.85	6.59	63	15	40.1	3.49			-4.8	0.003
1552	460375513	Fructose-Bisphosphate Aldolase 2	689	42872	46366	6.07	5.97	33	13	41	4.35			-4.44	0.0051
1557	460375513	Fructose-Bisphosphate Aldolase 2	433	42872	46216	6.07	6.12	12	7	20.3	1.13			-2.69	0.057
1564	460373309	Fructose-Bisphosphate Aldolase 2	835	42656	46316	8.15	7.46	48	13	41.1	5.38			-3.79	0.0061

												Drought Vs Cntr		Drought Rw Vs Cntr Rw	
Spot	NCBI code	Description	Mascot Score	$Mass_{th}$	$Mass_{ex}$	pI_{th}	pI_{ex}	Peptides	Unique Peptides	Coverage %	emPAI score	Ratio	T-test	Ratio	T-test
1567	460373309	Fructose-Bisphosphate Aldolase 2	663	42656	45916	8.15	7.54	20	12	31.6	2.85			-3.23	0.036
1570	460404664	Malate Dehydrogenase	294	45023	45023	8.14	7.72	6	6	16.8	0.61	2.08	0.011		
1574	460373374	Ferredoxin--Nadp Reductase. Leaf-Type Isozyme	525	40774	45817	8.37	6.79	10	9	29.6	0.85			-2.34	0.012
	460373820	Atp Synthase Gamma Chain	309	41752	45817	8.15	6.79	6	6	19.4	0.54				
1578	460373820	Atp Synthase Gamma Chain	486	41752	45718	8.15	7.23	13	10	26.3	1.17			-2.97	0.0052
	460373374	Ferredoxin--Nadp Reductase. Leaf-Type Isozyme	475	40774	45718	8.37	7.23	12	8	25.4	0.85				
1579	*460395539*	*Protochlorophyllide Reductase*	*450*	*43514*	*45767*	*9.27*	*9.53*	*8*	*7*	*19.5*	*0.64*				
1622	460365477	Malonyl-Coa-Acyl Carrier Protein Transacylase. Mitochondrial-Like	877	38298	44835	6.24	6.14	17	16	47.9	3.07			-1.82	0.0047
1626	460373374	Ferredoxin--Nadp Reductase. Leaf-Type Isozyme	398	40774	44835	8.37	6.26	9	8	26.8	0.7			-2.63	0.017
1519	460373374	Ferredoxin--Nadp Reductase. Leaf-Type Isozyme	535	40774	46467	8.37	6.94	12	9	32.9	1.63			-5.27	0.026
1619	460373374	Ferredoxin--Nadp Reductase. Leaf-Type Isozyme	532	40774	44689	8.37	6.40	10	9	28.2	1.02			-2.05	0.0033
1627	460373374	Ferredoxin--Nadp Reductase. Leaf-Type Isozyme	540	40774	44981	8.37	6.66	12	10	31.5	1.41			-3.3	0.0057
1629	460373374	Ferredoxin--Nadp Reductase. Leaf-Type Isozyme	540	40774	44689	8.37	6.78	15	11	38.4	1.88			-3.16	0.0081
1632	460373374	Ferredoxin--Nadp Reductase. Leaf-Type Isozyme	687	40774	44786	8.37	6.88	30	12	47	3.47			-4.22	0.00096
1633	460373374	Ferredoxin--Nadp Reductase. Leaf-Type Isozyme	740	40774	44786	8.37	7.02	24	15	42.5	3.09			-5.3	0.016
1634	460373374	Ferredoxin--Nadp Reductase. Leaf-Type Isozyme	638	40774	44786	8.37	6.54	32	12	37.6	2.43			-2.96	0.00053
1635	460373374	Ferredoxin--Nadp Reductase. Leaf-Type Isozyme	672	40774	44689	8.37	7.11	21	14	44.2	3.09			-3.89	0.0024
1636	460373374	Ferredoxin--Nadp Reductase. Leaf-Type Isozyme	617	40774	44544	8.37	7.19	21	11	30.9	1.63			-4.92	0.015
1641	460373374	Ferredoxin--Nadp Reductase. Leaf-Type Isozyme	1212	40774	44544	8.37	7.28	68	22	63.3	15.75			-7.01	0.0047
1642	460373374	Ferredoxin--Nadp Reductase. Leaf-Type Isozyme	842	40774	44399	8.37	7.39	54	15	48.9	5.36			-7.9	0.015
1643	460373374	Ferredoxin--Nadp Reductase. Leaf-Type Isozyme	771	40774	44159	8.37	7.73	35	15	48.3	2.43			-3.65	0.054
1650	460373374	Ferredoxin--Nadp Reductase. Leaf-Type Isozyme	708	40774	44159	8.37	7.54	23	14	47.2	2.43			-6.44	0.012
1659	460373374	Ferredoxin--Nadp Reductase. Leaf-Type Isozyme	551	40774	43873	8.37	8.29	14	10	32	1.41			-5.15	0.021
1660	460373374	Ferredoxin--Nadp Reductase. Leaf-Type Isozyme	764	40774	43921	8.37	7.85	17	15	53.3	3.09			-6.23	0.0068
1661	460373374	Ferredoxin--Nadp Reductase. Leaf-Type Isozyme	605	40774	43921	8.37	7.91	19	11	37.6	2.14			-7.49	0.027
1662	460373374	Ferredoxin--Nadp Reductase. Leaf-Type Isozyme	648	40774	43921	8.37	7.96	28	12	37.3	2.14			-8.33	0.044
1664	460373374	Ferredoxin--Nadp Reductase. Leaf-Type Isozyme	639	40774	43873	8.37	8.11	20	12	47.2	1.41			-6.7	0.016
1654	460373374	Ferredoxin--Nadp Reductase. Leaf-Type Isozyme	644	40774	44064	8.37	7.63	20	14	42.5	2.14			-7.4	0.0081
1704	83743301	Plastid Lipid Associated Protein Chrc	293	35685	41966	5.26	5.13	5	5	21.2	0.49	1.69	0.026		
1711	83743301	Plastid Lipid Associated Protein Chrc	424	35685	41966	5.26	5.21	13	9	30.1	1.47	1.81	0.02		
1709	460372520	Oxygen-Evolving Enhancer Protein 1	1014	35478	42194	5.84	5.38	30	18	59.2	5.78	-2.02	0.04		
	19157	33Kda Precursor Protein Of Oxygen-Evolving Complex	886	35154	42194	5.91	5.38	21	16	45.9	4.08				
1737	*460372520*	*Oxygen-Evolving Enhancer Protein 1*	*1000*	*35478*	*41514*	*5.84*	*5.49*	*24*	*16*	*57.7*	*4.54*				
	19157	*33Kda Precursor Protein Of Oxygen-Evolving Complex*	*780*	*35154*	*41514*	*5.91*	*5.49*	*18*	*13*	*41*	*2.75*				
1739	19157	33Kda Precursor Protein Of Oxygen-Evolving Complex	1368	35154	41290	5.91	5.61	70	24	66.9	18.06	1.58	0.019		
	460372520	Oxygen-Evolving Enhancer Protein 1	1335	35478	41290	5.84	5.61	64	24	64.3	17.55				
2013	*19157*	*33Kda Precursor Protein Of Oxygen-Evolving Complex*	*188*	*35154*	*32743*	*5.91*	*5.20*	*3*	*3*	*18.8*	*0.36*				
	460372520	*Oxygen-Evolving Enhancer Protein 1*	*219*	*35478*	*32743*	*5.84*	*5.20*	*4*	*4*	*21.6*	*0.35*				
1821	460385181	Gamma Carbonic Anhydrase 2. Mitochondrial-Like	233	29604	39239	6.8	8.60	6	6	29.3	1.06	-1.46	0.048		
1843	460402307	29 Kda Ribonucleoprotein A	819	31491	39491	4.67	4.67	34	13	57.2	6.67			-1.64	0.026
1906	6634469	Putative Ascorbate Peroxidase (Tl29)	730	37898	36531	8.27	9.22	27	15	47.2	5.04			-3.45	0.025
1907	*6634469*	*Putative Ascorbate Peroxidase (Tl29)*	*572*	*37898*	*36531*	*8.27*	*9.27*	*24*	*10*	*29.6*	*2.11*				
1972	460401859	Uracil Phosphoribosyltransferase-Like Isoform 1	232	31657	33716	8.54	7.35	4	4	17.1	0.57			-2.13	0.02
	56562177	Carbonic Anhydrase	163	34845	33716	6.67	7.35	5	4	15.9	0.36				
1976	56562177	Carbonic Anhydrase	430	34845	33825	6.67	7.96	13	10	34.9	1.05			-1.75	0.044
1977	460412635	20 Kda Chaperonin	848	26626	33862	8.55	6.65	51	14	61.9	8.64			-1.96	0.0005
1980	460412635	20 Kda Chaperonin	300	26626	33825	8.55	6.72	12	6	24.2	1.22			-1.56	0.043
1982	56562177	Carbonic Anhydrase	526	34845	33789	6.67	7.85	14	8	35.8	1.79			-3.38	0.032
1983	56562177	Carbonic Anhydrase	553	34845	33862	6.67	8.07	15	12	42.4	2.09			-3.54	0.023
1985	56562177	Carbonic Anhydrase	406	34845	34009	6.67	8.62	9	9	35.5	1.27			-5.79	0.024
1989	56562177	Carbonic Anhydrase	661	34845	33862	6.67	8.16	29	12	44.5	3.21			-6.13	0.0076
1991	56562177	Carbonic Anhydrase	407	34845	33899	6.67	8.34	8	8	29.9	1.05			-8.07	0.011
1992	56562177	Carbonic Anhydrase	534	34845	33899	6.67	8.40	11	8	28.7	1.52			-10.18	0.015
1993	56562177	Carbonic Anhydrase	487	34845	34046	6.67	8.46	13	10	38.9	1.79			-5.63	0.04
1736	460367599	50S Ribosomal Protein L1	582	37092	41156	9.32	9.71	14	13	36.1	1.89			2.45	0.018
2052	460368630	Upf0603 Protein At1G54780	111	31745	31286	6.85	5.69	3	3	12.1	0.4			-1.9	0.014
	115803	Chlorophyll A-B Binding Protein 4	86	28984	31286	5.33	5.69	2	2	11.7	0.28				
2057	460414390	Atp Synthase Delta Chain	126	27315	31422	8.9	5.13	2	2	9.2	0.14			-1.61	0.018
2060	460414390	Atp Synthase Delta Chain	417	27315	31286	8.9	5.37	15	6	33.6	1.83			-1.59	0.033
2125	460414390	Atp Synthase Delta Chain	578	27315	30089	8.9	5.38	25	7	35.6	2.67			-1.66	0.016
2046	19317	Photosystem Ii 23 Kda Protein	252	27946	31627	8.26	6.14	6	6	25.6	0.89	1.63	0.054		
2116	19317	Photosystem Ii 23 Kda Protein	409	27946	30351	8.26	5.59	12	9	31	1.43	2.24	0.041		
2140	19317	Photosystem Ii 23 Kda Protein	720	27946	29317	8.26	6.06	34	14	45	8.86	1.76	0.027		
2141	19317	Photosystem Ii 23 Kda Protein	548	27946	29412	8.26	6.16	31	12	50	5.73	1.81	0.033		
2142	19317	Photosystem Ii 23 Kda Protein	683	27946	29317	8.26	5.94	32	13	45	4.93	1.69	0.053		
2144	19317	Photosystem Ii 23 Kda Protein	615	27946	29222	8.26	5.59	19	12	47.7	4.93	2.13	0.0048		
2146	19317	Photosystem Ii 23 Kda Protein	711	27946	29348	8.26	5.82	69	12	42.6	10.19	1.76	0.036		

												Drought Vs Cntr		Drought Rw Vs Cntr Rw	
Spot	NCBI code	Description	Mascot Score	$Mass_{th}$	$Mass_{ex}$	pI_{th}	pI_{ex}	Peptides	Unique Peptides	Coverage %	emPAI score	Ratio	T-test	Ratio	T-test
2153	460407194	Elongation Factor P-Like	238	25499	28813	8.25	7.10	6	5	32.3	1.3	1.73	0.029		
2203	460400427	Thylakoid Lumenal Protein At1G12250	374	33826	26277	9.27	5.94	5	5	23	0.7			-1.82	0.045
	460367516	Thylakoid Lumenal 19 Kda Protein	310	26103	26277	5.96	5.94	9	6	17.8	1.26				
2215	*460405820*	*30S Ribosomal Protein S10*	*196*	*20831*	*26135*	*9.15*	*9.37*	*4*	*4*	*21.3*	*0.96*				
2257	*460365069*	*Peptidyl-Prolyl Cis-Trans Isomerase Cyp20-2*	*464*	*26690*	*26190*	*9.2*	*9.10*	*10*	*9*	*40.7*	*1.54*				
2341	84371989	Atp Synthase Cf1 Epsilon Subunit	531	14571	22824	5.43	6.78	16	10	63.2	12.75	-2.25	0.022		
2342	84371989	Atp Synthase Cf1 Epsilon Subunit	305	14571	22824	5.43	6.88	9	5	37.6	4.3	-1.97	0.0072		
2346	84371989	Atp Synthase Cf1 Epsilon Subunit	529	14571	22824	5.43	6.54	60	9	63.2	27.1	-1.69	0.0096	-2.78	0.033
2348	84371989	Atp Synthase Cf1 Epsilon Subunit	430	14571	22849	5.43	6.64	30	7	60.9	7.54	-1.81	0.012	-3.16	0.022
2349	84371989	Atp Synthase Cf1 Epsilon Subunit	417	14571	22799	5.43	6.70	15	6	48.9	9.83	-1.99	0.01		
2339	84371989	Atp Synthase Cf1 Epsilon Subunit	359	14571	22874	5.43	6.93	10	8	62.4	4.3	-1.92	0.0067		
2363	51457944	Photosystem Ii Oxygen-Evolving Complex Protein 3	282	24557	22432	9.64	9.52	20	5	25.7	1.38	1.84	0.028		
2394	51457944	Photosystem Ii Oxygen-Evolving Complex Protein 3	607	24557	21573	9.64	9.51	36	28	49.7	6.55	2.47	0.048		
2397	51457944	Photosystem Ii Oxygen-Evolving Complex Protein 3	285	24557	21597	9.64	9.61	7	5	28.7	1.06	2.09	0.0054		
2433	51457944	Photosystem Ii Oxygen-Evolving Complex Protein 3	176	24557	20636	9.64	9.50	5	4	20.9	0.33	1.96	0.0086		
2459	460397342	Thioredoxin M4	145	19843	19004	8.62	5.84	3	3	16.5	0.7			-3.19	0.031
2505	19334	Ribulose 1.5-Bisphosphate Carboxylase/Oxygenase	237	20446	17827	6.73	6.48	18	4	28.9	3.71			-5.99	0.018
2509	19334	Ribulose 1.5-Bisphosphate Carboxylase/Oxygenase	283	20446	17750	6.73	6.28	38	5	32.2	4.6			-6.21	0.0051
2511	19334	Ribulose 1.5-Bisphosphate Carboxylase/Oxygenase	541	20446	17731	6.73	6.03	53	10	48.9	12.25			-5.42	0.0059
2513	19334	Ribulose 1.5-Bisphosphate Carboxylase/Oxygenase	253	20446	17712	6.73	6.41	11	5	32.2	2.34			-5.39	0.029
2515	19334	Ribulose 1.5-Bisphosphate Carboxylase/Oxygenase	296	20446	17750	6.73	5.71	34	6	35.6	4.6			-4.19	0.021
2506	19334	Ribulose 1.5-Bisphosphate Carboxylase/Oxygenase	427	20446	17827	6.73	5.84	16	9	45.9	6.82			-3.93	0.00097
2516	19334	Ribulose 1.5-Bisphosphate Carboxylase/Oxygenase	380	20446	17559	6.73	5.49	33	8	45.9	6.82			-3.59	0.0094
2555	19300	Plastocyanin	182	17039	17039	5.06	5.06	9	3	27.1	2.41			-2.22	0.0035
2005	*7331143*	*Chaperonin 21 Precursor*	*199*	*26603*	*33244*	*6.85*	*5.68*	*4*	*4*	*19*	*0.7*				
2008	*7331143*	*Chaperonin 21 Precursor*	*419*	*26603*	*33136*	*6.85*	*5.59*	*7*	*7*	*37.5*	*1.54*				
2375	460365655	Phosphatidylglycerol/Phosphatidylinositol Transfer Protein-Like Isoform 1	99	22075	22022	5.92	7.76	3	2	7.7	0.61	2.35	0.019		
2468	460365655	Phosphatidylglycerol/Phosphatidylinositol Transfer Protein-Like Isoform 1	132	22075	19253	5.92	7.75	2	2	13.8	0.38			4.03	0.044
1760	460387893	Chlorophyll B Reductase Nol	138	38712	40889	9.22	9.20	3	3	9.1	0.32	1.7	0.001		
1793	460387893	Chlorophyll B Reductase Nol	88	38712	39624	9.22	9.49	2	2	7.7	0.1			1.62	0.02
2421	38231570	Pii-Like Protein	177	21775	20974	9.33	6.55	5	4	22.6	0.91	-1.61	0.013	-2.61	0.006
2409	75266239	Constitutive Plastid-Lipid Associated Protein	193	20056	21203	8.81	5.85	4	4	25.7	1.01			-1.62	0.022
2411	75266239	Constitutive Plastid-Lipid Associated Protein	160	20056	21203	8.81	5.76	4	3	13.9	0.69			-2.02	0.033
2412	19193	Superoxide Dismutase [Cu-Zn]	198	22328	21065	5.77	6.29	5	3	22.1	0.88			-2.12	0.0015
2386	460378455	Thylakoid Lumenal 17.4 Kda Protein	126	25712	21832	6.71	5.26	2	2	10.3	0.32			-1.91	0.049
2396	460397890	Peptidyl-Prolyl Cis-Trans Isomerase Fkbp13	115	24353	21597	8.69	9.25	2	2	6.6	0.34	1.77	0.048		
2049	15485610	Clp Protease 2 Proteolytic Subunit	302	32577	31834	8.78	6.64	7	6	24.1	0.73			1.69	0.0052
2084	84372035	Photosystem I Subunit Vii	148	9545	30983	6.67	7.26	3	3	39.5	1.88	1.87	0.04		
2095	84372035	Photosystem I Subunit Vii	125	9545	30483	6.67	9.08	2	2	9.5	1.03	2.33	0.043		
2099	*460396303*	*Auxin-Binding Protein Abp19A-Like*	*147*	*22188*	*30417*	*6.25*	*8.23*	*2*	*2*	*32.1*	*0.37*				
2104	84372035	Photosystem I Subunit Vii	133	9545	30384	6.67	9.12	2	2	32.1	1.03	2.19	0.016		
2105	84372035	Photosystem I Subunit Vii	196	9545	30384	6.67	9.16	3	3	39.5	1.88	2.47	0.019		
2139	84372035	Photosystem I Subunit Vii	109	9545	29540	6.67	7.97	2	2	32.1	1.03	3.00	0.062		
2023	19182	Type Iii Chlorophyll A/B-Binding Protein	202	29344	32566	8.96	7.41	5	5	20.1	0.28	1.55	0.065		
2041	460392422	Psbp Domain-Containing Protein 6	125	27450	31937	8.35	8.06	3	3	16	0.48			2.03	0.024
2075	19182	Type Iii Chlorophyll A/B-Binding Protein	127	29344	30983	8.96	7.15	3	3	13.2	0.28	2.25	0.028		
2091	460392422	Psbp Domain-Containing Protein 6	84	27450	30815	8.35	7.81	2	2	12	0.3	1.96	0.06		
